# Computational Studies on Selected Macrolides Active against *Escherichia coli* Combined with the NMR Study of Tylosin A in Deuterated Chloroform

**DOI:** 10.3390/molecules27217280

**Published:** 2022-10-26

**Authors:** Biljana Arsic, Jill Barber, Ana Cikos, Manikandan Kadirvel, Emilija Kostic, Andrew J. McBain, Jelena Milicevic, Angela Oates, Andrew Regan

**Affiliations:** 1 Division of Pharmacy and Optometry, School of Health Sciences, University of Manchester, Oxford Road, Manchester M13 9PT, UK; 2 Department of Chemistry, Faculty of Sciences and Mathematics, University of Nis, Visegradska 33, 18000 Nis, Serbia; 3 NMR Centre, Ruđer Bošković Institute, Bijenička Cesta 54, 10000 Zagreb, Croatia; 4 Faculty of Medicine, University of Nis, Bulevar dr Zorana Djindjica 81, 18000 Nis, Serbia; 5 Laboratory for Bioinformatics and Computational Chemistry, Vinca Institute of Nuclear Sciences, National Institute of the Republic of Serbia, The University of Belgrade, Mike Petrovica Alasa 12-14, 11351 Belgrade, Serbia; 6 Centre for Biomedicine, Hull York Medical School, Faculty of Health Sciences, University of Hull, Hull HU6 7RX, UK; 7 Department of Chemistry, University of Manchester, Oxford Road, Manchester M13 9PL, UK

**Keywords:** macrolides, bacteria, 2D ROESY NMR

## Abstract

Although many antibiotics are active against Gram-positive bacteria, fewer also show activity against Gram-negative bacteria. Here, we present a combination of in silico (electron ion-interaction potential, molecular docking, ADMET), NMR, and microbiological investigations of selected macrolides (14-membered, 15-membered, and 16-membered), aiming to discover the pattern of design for macrolides active against Gram-negative bacteria. Although the conformational studies of 14-membered and 15-membered macrolides are abundant in the literature, 16-membered macrolides, and their most prominent representative tylosin A, have received relatively little research attention. We therefore report the complete ^1^H and ^13^C NMR assignment of tylosin A in deuterated chloroform, as well as its 3D solution structure determined through molecular modelling (conformational search) and 2D ROESY NMR. Additionally, due to the degradation of tylosin A in deuterated chloroform, other species were also detected in 1D and 2D NMR spectra. We additionally studied the anti-bacterial activity of tylosin A and B against selected Gram-positive and Gram-negative bacteria.

## 1. Introduction

Macrolide antibiotics have been proven as effective, anti-bacterial agents [1], some active against both Gram-positive and Gram-negative bacteria. However, searching for macrolides active against Gram-negative bacteria is a difficult task and is not a systematic process. Here, we attempted to identify key features (parameters) to facilitate future searches for macrolides specifically active against Gram-negative bacteria. The starting point was erythromycin A, a 14-membered macrolide antibiotic produced by *Saccharopolyspora erythraea*, showing a broad spectrum of antibacterial activity against Gram-positive bacteria [2]. This compound also exhibits anti-malarial activity against *Plasmodium* species in vitro and in vivo*,* both alone [3,4,5,6] and particularly in the combination with artemisinin [7]. Its weakest point, acid sensitivity [8], has inspired numerous approaches to address the issue. The successful attempts include flurithromycin, (8*S*)-8-fluoroerythromycin A [9], and the most famous clarithromycin [10] and azithromycin [11,12,13,14,15], although some others, such as erythromycin B, showed a better acid stability profile while retaining a similar anti-bacterial spectrum [16]. Erythromycin C structure was solved in organic and aqueous solutions [17], and it is one of the modifications of erythromycin A. Its antibacterial activity is less for most of the tested bacteria compared to erythromycin A and B [17,18], presumably due to also the presence of the large proportion of hemiacetal relative to erythromycin A and B [17]. 

Unlike erythromycin A, its semi-synthetic cousin azithromycin is generally effective against Gram-negative bacteria, while its numerous derivatives show even more improved activity [19]. 

Tylosin A, a 16-membered macrolide antibiotic, has so far only been used in veterinary medicine against Gram-positives [20] and is produced by several *Streptomyces* species including *S. fradiae* [21], *S. rimosus* [22], and *S. hygroscopicus* [23]. Out of those, only *S. fradiae* is used in industrial production [24]. Different tylosin A and tylosin B (a product of the acid degradation of tylosin A) derivatives were synthesized [25,26], and some show better activity against Gram-negative bacteria than tylosin A. 

Full NMR assignments of tylosin A in phosphate-buffered deuterated water and tylosin B in CDCl_3_ were presented by Arsic et al. (2017) [27]. It was the first complete assignment of all signals observed in ^1^H and ^13^C spectra. Earlier, Rossi et al. (1992) [28] and Simova and Ivanova (1996) [29] reported spectral assignments of tylosin A in aprotic solvents (CDCl_3_ and CD_3_CN). Previously, computationally modelled 3D structures of tylosin A in chloroform were reported by Simova and Ivanova (1996) [29] and Ivanov (1998; 2002) [30,31]. Due to its dielectric constant, chloroform can be used to mimic the conditions of the target active site [32,33], which was crucial for our docking and target interactions approach. 

Published data on macrolides interacting with *Escherichia coli* ribosomes show that both erythromycin A and tylosin A bind in the same active site through a similar two-step process. The first step is fast, involving a low-affinity binding site situated at the entrance of the exit tunnel of the large ribosomal subunit, binding primarily through hydrophobic portions. The second step follows through slow conformational changes managed by the antibiotic hydrophilic portion which pushes the drug deeper into the tunnel towards a high-affinity site [34]. Tylosin A, compared to erythromycin A, shifts to the high-affinity site more rapidly because of the additional interaction of the mycinose sugar with the loop of H35 in domain II of 23S rRNA [34]. Using *E. coli* as the binding target, we checked the docking sites and the affinity of macrolides additional to tylosin A and erythromycin A (and more active against Gram-negative bacteria) to *E. coli*. Useful conclusions drawn from EIIP (electron-ion interaction potential) screening, molecular docking, and ADMET studies were envisioned to help in the creation of new more effective macrolides active against Gram-negative bacteria. 

The rationale behind in silico approaches is the relatively lower cost and the time factor involved, when compared to standard experimental approaches for ADMET profiling [35]. As an example, it only takes a minute in an in-silico model to screen 20,000 molecules, but takes 20 weeks in the “wet” laboratory to do the same exercise [36]. Therefore, using in silico models to predict favorable ADME characteristics helps to overcome one of the most daunting hurdles for drug development. Unfortunately, due to the macrolide size and complexity, creating a trustworthy prediction model is much more complicated than in the case of small pharmaceuticals.

## 2. Results and Discussion

### 2.1. NMR Studies

A full NMR analysis including extraction of chemical shifts, coupling constants, and HMBC connectivities (Table 1), as well as an analysis of NOE interactions (Table S1), were performed on tylosin A, aiming to obtain structural data needed for conformational analysis. The strategy for the assignment follows those previously reported [27]. Data were collected from 1D and 2D NMR techniques (^1^H, ^13^C, DEPT 135, 2D COSY, 2D HMQC, 2D HMBC, 2D ROESY) (Figures S1–S7 in Supplementary Material). 

**Figure 1:**
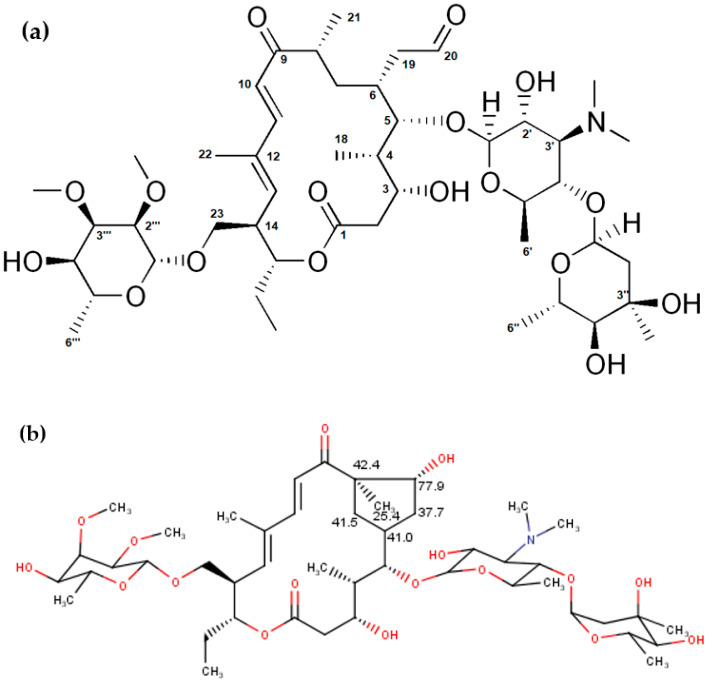
(**a**) Structure of tylosin A and (**b**) predicted ^13^C NMR chemical shifts characteristic for the structure of tylosin aldol.

Although the chemical shifts and coupling constants for tylosin A fit well with the previously published data [29], our analysis showed some additional peaks (two CH_2_ and two CH signals with carbon shifts around 40 ppm, methyl at 25 ppm, and one CH with carbon at 77 ppm) which can be attributed to the degradation products of tylosin A, such as tylosin aldol. These values are in good agreement with the expected values for carbon chemical shifts from an NMR predictor (Figure 1, [37]). Additionally, they correspond well to the previously reported tylosin aldol degradant [38]. Proton chemical shifts could not be compared due to the large overlap in the NMR spectra, as well as low accuracy of proton prediction. 

The challenges in the assignment did not only come from the degradation products, but also because of some special features (Figure S6—unexpected extra signals in HMQC where the presence of ^13^C leads to strong ^1^H-^1^H coupling (i.e., where one or both of the ^13^C satellites in the ^1^H spectrum is strongly coupled because the chemical shift difference between two protons is roughly equal to half the one-bond ^13^C-^1^H coupling constant) were observed [39]). This effect in heteronuclear chemical shift correlation 2D NMR spectra is called “virtual one-bond coupling” [39]. 

### 2.2. Computational Studies

#### 2.2.1. EIIP Screening

The number of valence electrons and W (the main energy term of valence electrons) are essential physical parameters for the estimation of long-range properties of biological molecules [40]. It was also previously demonstrated that there is a strong correlation between W and biological properties of the investigated molecules (mutagenicity, carcinogenicity, etc.) [41,42]. 

Here, we use W to determine the values of active compounds against Gram-negative bacteria, to give directions for the design of compounds that should be active against Gram-negative bacteria (Table 2).

W is a good predictor, but caution must be taken because different classes of the compounds, although having the same target, can have very similar values of W and quite different activities against bacteria. Therefore, we formed conclusions only within the particular group of macrolides. Erythromycin A, B, and C have activities against Gram-positive bacteria, but almost no activity against Gram-negative bacteria. Their values for W are 0.0927, 0.0949, and 0.0913 Ry, respectively. Therefore, 14-membered macrolides with these W values or quite similar would not possess the activity against Gram-negative bacteria. The investigated 15-membered macrolides (**7** and **8**) with good activities against Gram-negative bacteria have W values equal to 0.0762 and 0.0767 Ry, respectively. So, 15-membered macrolides with these W values or quite similar will have activities against Gram-negative bacteria. For the 16-membered macrolide antibiotics, tylosin A and B, having similar and negligible activities against both Gram-positive and Gram-negative bacteria, possess similar W values: 0.0812 and 0.0821 Ry, respectively. Therefore, 16-membered macrolides with these and similar values would not possess significant activities against Gram-negative bacteria. From this class of macrolides, only derivatives with W values of 0.0841, 0.0806, 0.0765, 0.0739, 0.0731, 0.0909 Ry (Table 2) will have significant activities against Gram-negative bacteria. 

#### 2.2.2. Conformational Analysis of Selected Macrolide Antibiotics 

The unconstrained conformational search was performed on selected 14-membered, 15-membered, and 16-membered macrolides using chloroform as a solvent. 

The searches for all the selected 14-membered macrolides (erythromycin A, erythromycin B, and erythromycin C) were carried out using the AMBER^*^ force field [43,44,45,46,47]. Erythromycin A global minimum (E = 58.66 kJ/mol, found five times) is a folded-in structure, erythromycin B global minimum (E = 102.84 kJ/mol, six times) is a folded-out structure, and erythromycin C global minimum (E = 53.59 kJ/mol, 24 times) is a folded-in structure [48]. Because the biologically active conformation of erythromycin A and B is a folded-out structure [16,49] and we assume that it is the same situation for erythromycin C, we performed constrained conformational searches of erythromycin A and C (H4–H11 2.5 ± 0.3 Å, H5–H18 2.5 ± 0.3 Å), and obtained the folded-out structure for erythromycin A with E = 62.27 kJ/mol (three times), and for erythromycin C with E = 55.73 kJ/mol (eight times). 

Two 15-membered macrolides were taken for conformational analysis using AMBER^*^ force field: 3-*O*-Descladinosyl-3-*O*-[2-(2-pyridyl)acetyl]-6-*O*-(3-{2-[(3-carboxy-6-fluoro-1-cyclopropyl-1,4-dihydro-4-oxoquinolin-7-yl)piperazin-1-yl]}propanoyl)azithromycin (**7**), 3-*O*-Descladinosyl-3-*O*-[2-(2-pyridyl)acetyl]-6-*O*-(3-{2-[3-(3-carboxy-6-fluoro-8-methoxy-1-cyclopropyl-1,4-dihydro-4-oxoquinolin-7-yl)methylpiperazin-1-yl]}propanoyl)azithromycin (**8**). Derivative **7** shows a global minimum with E = 171.01 kJ/mol repeated three times, and derivative **8** global minimum with E = 229.48 kJ/mol appearing only once. Because the folded-out conformation did not appear among the ten lowest-energy conformations in the unconstrained conformational search in either case, we performed the constrained conformational search with the distances constrained [H4–H11 (2.5 ± 0.3 Å), H5–H18 (2.5±0.3 Å)]. The folded-out structure as a global minimum for derivative **7** has an energy equal to 241.37 kJ/mol, and for derivative **8**, E = 255.22 kJ/mol. 

For 16-membered macrolide tylosin B, the conformational analysis was performed in chloroform with the AMBER^*^ force field. The global minimum using the AMBER^*^ force field was found only twice with the energy E = 84.98 kJ/mol. For tylosin A, the conformational analysis with an MM2 force field [50] used before [27] gave the global minimum with the energy E = −716.22 kJ/mol and appeared seven times, and with the AMBER^*^ force field, only two times with the energy E = 108.94 kJ/mol. Rosaramicin gave a global minimum eight times using the AMBER^*^ force field with the energy E = 38.24 kJ/mol, and tildipirosin gave a global minimum only once with the energy E = 55.48 kJ/mol. The global minima of 20-deoxy-20-{*N*-methyl-*N*-[1-(2-naphthyl)-1*H*-1,2,3-triazol-4-yl]methylamino}-5-*O*-mycaminosyltylonolide (**1**) repeated twice with the energy 113.48 kJ/mol; 20-deoxy-20-{*N*-methyl-*N*-[1-(6-quinolyl)-1*H*-1,2,3-triazol-4-yl]methylamino}-5-*O*-mycaminosyltylonolide (**2**) only once with the energy 101.41 kJ/mol; 20-deoxy-20-{*N*-methyl-*N*-[1-(3-quinolyl)-1*H*-1,2,3-triazol-4-yl]methylamino}-5-*O*-mycaminosyltylonolide (**3**) four times with the energy 121.28 kJ/mol; 20-deoxy-20-{*N*-methyl-*N*-[1-(3-quinolyl)-1*H*-1,2,3-triazol-4-yl]methylamino}-5-*O*-mycaminosyltylonolide (**4**) five times with the energy 127.21 kJ/mol; 20-deoxy-20-{*N*-benzyl-*N*-[1-(3-quinolyl)-1*H*-1,2,3-triazol-4-yl]methylamino}-5-*O*-mycaminosyltylonolide (**5**) only once with the energy 129.99 kJ/mol; and 20-deoxy-20-{*N*-methyl-*N*-[1-(1-naphthyl)-1*H*-1,2,3-triazol-4-yl]methylamino}-5-*O*-mycaminosyltylonolide (**6**) fifteen times with the energy 156.27 kJ/mol.

#### 2.2.3. Prediction of the Solution Structures of Tylosin A in Chloroform

The results of the molecular modelling of tylosin A in chloroform using the software MacroModel and conformational search (MM2 and AMBER^*^ force fields) were in good accordance with the distances observed in the 2D ROESY NMR spectrum (Tables S1 and S2 in the Supplementary Material). The discrepancies were observed in the global minimum obtained with the MM2 force field with H10-H_3_22, H11-H13, H13-H11, H14-H_3_22, H1¨-H_3_6¨, and H_3_6‴-1‴. The mispredictions of the molecular modelling were previously observed for tylosin A, but were observed in water for H11-H13 using the same force field-MM2 [27]. Less accurate predictions for contacts with some methyl groups can be explained by the free rotations of methyl groups requiring less energy. The AMBER^*^ force field shows better predictive character. ROESY interactions were in excellent agreement with the molecular modelling with the AMBER^*^ force field. Only H1¨–H_3_6¨ and H_3_6‴-1‴ were not predicted well, but it is not a big issue because of the rotation of methyl groups, which does not require large amount of energy. 

#### 2.2.4. Molecular Docking Studies

**Figure 2:**
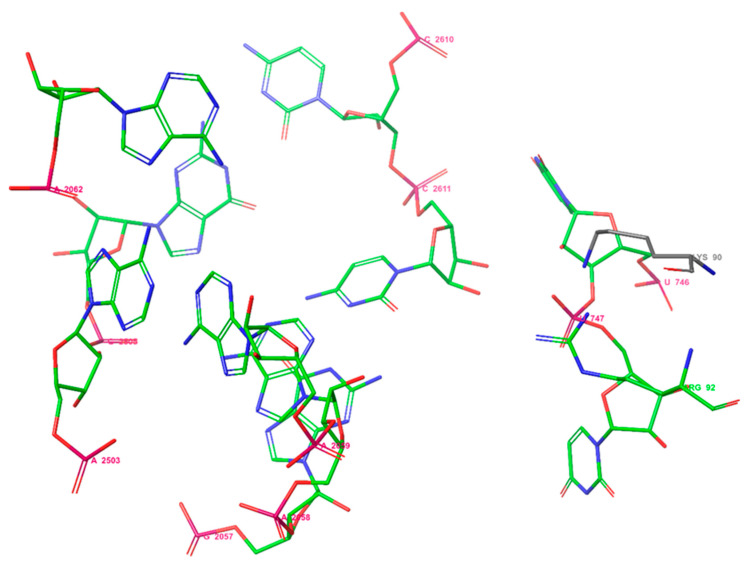
The binding site of erythromycin A in the *E. coli* ribosome.

Previously, it was shown that the binding site of erythromycin A to *E. coli* ribosomes consists of two amino acids: Lys90 and Arg92, and the nucleotides U746, U747, A2062, A2503, G2505, G2057, A2058, A2059, C2611, and C2610 [51] (Figure 2). So, selected macrolides were docked into proteins of the ribosome from *E. coli* considering the amino acids Lys90 and Arg92 using Glide under Schrodinger 2021-2. 

It was shown that the molecular docking into the narrow region around amino acids Lys 90 and Arg 92 was possible only for 15-membered macrolides and 16-membered macrolides except for rosaramicin and tylosin A and B. The docking scores together with corresponding energies are available in Table 3 (energies, E, are expressed in kcal/mol). Lower values of docking score mean a higher ability to interact with the target. Based on this statement, we can say that **3** will have the highest affinity amongst the selected macrolides to interact with the target, and the lowest ability will, therefore, be shown by **7**. 

Graphical representations of the interactions between the selected macrolides and the target are available in the Supplementary Material (Figures S8–S16). 

All selected macrolides were also docked into the nucleotide segment of the binding site using Patchdock. Macrolide **3** has a solution with the lowest energy equal to −64.17, and it shows mainly interactions with residues A2503, G2505, and A2059 through van der Waals, a conventional hydrogen bond (A2503), Pi-alkyl, Pi-Pi T-shaped, Pi-lone pair, a carbon-hydrogen bond, and a Pi-Donor hydrogen bond (Figure S17). Macrolide **5** shows an energy value of −70.07, and has more interactions compared to **3** with residues A2058, A2062, A2503, G2505, C2611, C2610, and A2059 with two conventional hydrogen bonds among other present interactions (Figure S18). Macrolide **6**, with a global energy of −68.94, shows plenty of interactions similarly to **5**; conventional hydrogen bonds like in **5** were with residues A2503, A2062, and other interactions with residues G2505, C2610, A2503, A2059, and A2508 (Figure S19). In the case of **2**, the solution with the highest score (5918) was also the structure after the refinement with the lowest global energy (−70.96), and it shows only interactions with the nucleotides A2062, C2611, G2505, and C2610, with two conventional hydrogen bonds with A2062 and C2611 among other interactions (Figure S20). Macrolide antibiotic **4** shows the lowest energy of −60.16 in the complex, and interactions with the residues G2057, U746, C2610, A2059 (conventional hydrogen bond), A2503, C2611, G2505, and an unfavourable bump with A2058 (Figure S21). Macrolide **1** shows that the global energy of a complex equals −72.67, and the interactions with A2059, A2062, C2611, G2505, A2503, G2505, and A2058 occur with no conventional hydrogen bond (Figure S22). Rosaramicin in the complex with the nucleotide segment of the binding site (global energy −55.08) has interactions (only a carbon-hydrogen bond, Pi-sigma, and Pi-alkyl) with the nucleotides A2059, A2062, C2610, and A2503 (Figure S23). 

Macrolide **8** shows, in the complex (the global energy −61.97), the interactions with the following residues: C2611, U746, A2062, A2058, and G2505, with no conventional hydrogen bonds and two unfavourable bumps with C2610 and C2611 (Figure S24). Macrolide **7** in the complex with the global energy of −70.33 shows various interactions with the nucleotides A2062 (conventional hydrogen bond), G2505, C2610, A2062, A2503, A2059, and A2058 (Figure S25). 

Erythromycin A docked into the RNAs of entry 4V7U shows interactions with the nucleotides not found in the binding site [51] and numerous unfavourable bumps. This simple in silico experiment tells us that the involvement of the amino acids in the binding of erythromycin A is very important. Molecular docking of erythromycin A into the nucleotide segment of the binding site (global energy of −51.95; the lowest energy complex is also the best docked complex-score equal to 5062) gives information about its interactions with the nucleotides G2057, C2611, and G2505 (conventional hydrogen bonds), and then A2058, C2610, A2059, and A2503 (Figure S26). Similarly to erythromycin A, erythromycin B in the complex with the nucleotides from the binding site shows the lowest global energy equal to −54.35, which is also the complex with the highest dock score, which is 5066. It shows that erythromycin B has interactions with A2058, G2057, and C2611 (conventional hydrogen bonds), and also interactions with C2610, A2062, A2503, G2505, and C2610 (Figure S27). Erythromycin C has interactions with the nucleotides within the binding site (in the complex with the global energy −54.45): C2610, G2505, C2611, and A2058, and one of them is an unfavourable bump (Figure S28). The lowest energy-refined complex of tylosin A with the binding site (energy equals to −59.73) shows interactions with G2505 and C2610 through the conventional hydrogen bonds, and other interactions with A2058, A2059, and A2503 (Figure S29). Tylosin B in the complex with the binding site (global energy equals −44.87) shows no hydrogen bonding interactions, but shows others through contact with U746, C2611, G2505, A2503, A2058, and A2059 (Figure S30). 

#### 2.2.5. ADMET Studies

The drug-likeness of compounds was assessed according to Lipinski’s rule of five [52], which considers molecular weight (<500 Da), the number of hydrogen-bond acceptors (≤10) and donors (≤5), and the octanol/water partition coefficient (≤5); and Jorgensen’s rule of three [53], which regards logS (>−5.7), PCaco (>22 nm/s), and primary metabolites (PM) (<7). The violations of these rules are essential for the optimization of biologically active compounds and should not be more than 1.

Table 4 presents the predicted ADMET properties of all compounds, and this table contains the following parameters: molecular weight (MW), number of rotatable bonds (RB), dipole moment (DM), molecular volume (MV), number of hydrogen donors (DHB), number of hydrogen acceptors (AHB), polar surface area (PSA), octanol/water partition coefficient (log P), aqueous solubility (log S), apparent Caco-2 cell permeability (PCaco), number of likely primer metabolic reactions (PM), percentage of human oral absorption (HOA (%)), and the violations of rules of three (VRT) and five (VRF). The theoretical calculations of ADME parameters are presented in Table 4 along with the violations of Lipinski’s and Jorgensen’s rules. According to these data, only rosaramicin has VRF = 1, and can be considered as a good oral drug candidate. Considering values for VRT, besides rosaramicin (VRT = 0), the drugs already known such as erythromycin A, B, and C, and tylosin A and B, are oral drugs (VRT = 1). In case of macrolides, however, Lipinski’s rule of five and in silico ADMET analysis with commercial software should be taken with some reserve, and models and rules should be developed separately [54,55,56] due to their frequent interaction with cellular membrane transporters [57,58,59] and chameleon-like conformational flexibility [54]. It was suggested earlier that the design of novel macrolides should be aimed at the optimization of the macrolide core (macrolactone entity with sugars and similar size constituents) with no large substituents and flexible linkers [60].

ToxTree was used to classify substances of toxicological hazard. According to The Cramer classification scheme (decision tree) used to estimate the Threshold of Toxicological Concern (TTC), all investigated substances are classified into Class III (most severe hazard). Toxicity profiles of compounds (test and control (azithromycin and artemisinin)) were predicted using ADMETLab 2.0. The results are presented in Table 5. ADMETLab descriptors for toxicity are hERG blockers, human hepatotoxicity (H-HH), drug-induced liver injury (DILI), mutagenicity (Ames tox), skin sensitivity (Skin Sens), eye irritation, and eye corrosion. Cellular assays on nuclear receptor signaling pathways (NR) and stress response pathways (SR) (Tox21) were also evaluated using ADMETLab 2.0. All investigated substances, except erythromycin A, B, and C, block hERG channels, which indicates their cardiotoxic potential. Hepatotoxic effects (calculated parameters for human hepatotoxicity and drug-induced liver injury) are predicted for all structures. All structures except erythromycin and artemisinin have the potential for respiratory toxicity. Only artemisinin has genotoxic potential, according to results of AMES test prediction, while artemisinin and rosaramicin both have carcinogenic potential. Except for artemisinin, tildipirosin, and tylosin A and B, the rest of the structures were not predicted to have skin irritation effects. Only artemisinin has eye corrosion and eye irritation potential. Assays such as P53 DNA damage, ATAD5 (ATPase family AAA domain-containing protein 5) assays (genotoxicity and mutagenicity) and stress response panel, and androgen and estrogen receptor signaling pathways assays (carcinogenicity) provide important information on toxicological properties. Erythromycin C, tylosin B, and compounds **6**–**8** interact with the full length of the androgen receptor, while artemisinin interacts only with the ligand-binding domain of the androgen receptor. Only tylosin A interacts with aromatase. All of the analysed compounds interact with the full length of the estrogen receptor, while artemisinin, rosaramicin, and tylosin A and B only interact with the ligand-binding domain. Results of the prediction of impact on ARE have shown that all compounds, except azithromycin, erythromycin, artemisinin, rosaramicin, can interfere with ARE, and eventually cause oxidative stress. Mitochondrial membrane potential (MMP) parameters of the compounds predicted showed that erythromycin A, tylosin A, and 20-deoxy-20-{*N*-methyl-*N*-[1-(1-naphthyl)-1*H*-1,2,3-triazol-4-yl]methylamino}-5-*O*-mycaminosyltylonolide showed mitochondrial toxicity, which compromises cell energy production. P53 is a tumour suppressor protein, activated following cellular insult, including DNA damage and other cellular stresses. Artemisinin, erythromycin A, and tylosin A can activate P53. None of the compounds showed potential interactions with nuclear receptors: PPAR-gamma receptor (glitazone receptor) involved in the regulation of glucose and lipid metabolism and AHR receptor (aryl hydrocarbon receptor), which mediates cellular responses to environmental pollutants. Additionally, none of the compounds has the potential to interfere with stress response pathways: ATAD5 is activated when a cell detects damage, and HSE (hear shock factor response element) is involved in the cell’s reaction to heat shock. For the classification endpoints, the prediction probability values are transformed into six symbols: 0–0.1 (---), 0.1–0.3 (--), 0.3–0.5 (-), 0.5–0.7 (+), 0.7–0.9 (++), and 0.9–1.0 (+++).

Using Pro Tox II, LD_50_ values and toxicological class were calculated. Rosaramicin has the minimal predicted LD_50_ value (1000 mg/kg). All compounds from series 2 have an LD_50_ value of 5000 mg/kg. All compounds are class 4 (harmful if swallowed: 300 < LD_50_ ≤ 2000) and class 5 (may be harmful if swallowed: 2000 < LD_50_ ≤ 5000). 

Based on the performed analysis using commercially available medicines (azithromycin and artemisinin) as controls, we can conclude that all investigated compounds can be used with the caution similarly shown to other commercially available medicines.

### 2.3. Determination of the Minimum Inhibitory Concentrations of Tylosin A and B

The minimum inhibitory concentrations of tylosin A and B against Gram-positive and Gram-negative bacteria are compared in Table 6. Although both tylosin A and tylosin B have antibacterial activity, this is lower or similar compared with clarithromycin and azithromycin. This suggests that the widespread use of tylosin in veterinary medicine owes much to the large doses used (infection may be treated by adding tylosin to the animals’ drinking water) or to the antiprotozoal and anti-inflammatory activities of tylosin.

All MICs were determined in triplicate. 

Literature values of MICs of other selected macrolides (**1**–**8**, tildipirosin, rosaramicin) are presented in Table 7.

## 3. Materials and Methods

### 3.1. NMR Analysis of Tylosin A

Tylosin A tartrate was obtained from Aldrich and used without further purification. All measurements were carried out in deuterated chloroform at 20 °C. The measurements were performed using Bruker Avance 400 MHz and 500 MHz NMR spectrometers (Karlsruhe, Germany). 

#### Acquisition and Processing Parameters for NMR Experiments

For the ^13^C{^1^H} spectrum, the operating frequency was 125.77 MHz, the P1 = 8.80 μs, AQ = 1.1 s, 2 dummy scans were used, SWH = 29,761.904 Hz, TD = 65,536; for the DEPT 135 spectrum, the operating frequency was 125.77 MHz, P1 = 8.80 μs, AQ = 1.1 s, dummy scans = 4, SWH = 29,761.904 Hz, TD = 65,536. An exponential function was used as a window function for processing the ^13^C spectrum with LB = 1.50 Hz. The same window function was used for DEPT 135 processing, but the LB value was 1.00 Hz. In the case of the COSY spectrum, the operating frequency was 500.132 MHz, AQ = 0.33 s, SWH = 6250.0 Hz, TD = 4096 × 256, and QSINE window functions using a Fourier number (SI) of 8192 and 2048 for the direct and indirect dimensions were performed, respectively, with SSB = 1. The acquisition parameters for the HSQC spectrum were AQ = 0.16 s, SW (F1) = 240.0 ppm, SWH = 6250.0 Hz, TD = 2048 × 256. The Echo-Antiecho procedure was used to produce phase-sensitive data (FnMODE) with operating frequencies for ^1^H and ^13^C equal to 500.132 MHz and 125.77 MHz, respectively. The processing parameters for HMQC were: window function-QSINE, SI = 4096 × 1024, SSB = 2. Regarding the HMBC, the acquisition parameters were: AQ = 0.33 s, SW(F1) = 240.0 ppm, SWH = 6250.0 Hz, TD = 4096 × 256, with operating frequencies for ^1^H and ^13^C equal to 500.13 MHz and 125.77 MHz, respectively. The ROESY experiments were recorded using the following acquisition parameters: operating frequency 500.13 MHz, TD = 2048 × 256, AQ = 0.16 s, D1 = 2 s, P15 = 200 ms, FnMODE-Echo-Antiecho; and the following processing parameters: SI = 4096 × 1024, the window function was QSINE, SSB = 1. 

### 3.2. Computational Studies

#### 3.2.1. EIIP Screening

The prediction that certain organic molecules can be targets for the specific receptors (long-range interactions: interactions at distances at >5 Å) is possible using the molecular descriptor “general model pseudopotential” (W) [40,41,42,62,63]:

W = 0.25(Z^*^/(2π))Sin(1.04πZ^*^),

where Z^*^ is the average quasi-valence number determined by:

$$Z^{*}=\frac{1}{N}\overset{m}{\underset{i=1}{\sum }}n_{i}Z_{i}$$

where *Z_i_* is the valence number of the *i*th atomic component, *n_i_* is the number of atoms of the *i*th component, *m* is the number of atomic components in the molecule, and *N* is the total number of atoms. W and Z^*^ values are expressed in Rydberg units (Ry).

#### 3.2.2. MacroModel Calculation Settings

**Figure 3:**
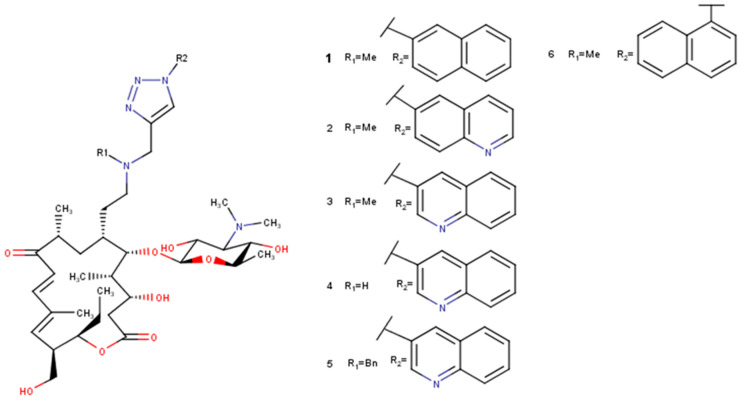
Selected derivatives **1**–**6** of 5-*O*-mycaminosyltylonolide.

**Figure 4:**
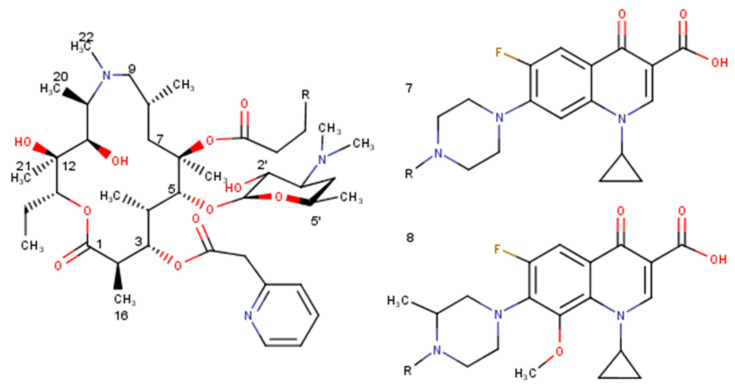
Selected derivatives of azithromycin (**7**, **8**).

An unconstrained conformational analysis of selected macrolide antibiotics—14-membered macrolides (erythromycin A, erythromycin B, erythromycin C), 16-membered macrolides (rosaramicin, tildipirosin, tylosin A, tylosin B, 20-deoxy-20-{*N*-methyl-*N*-[1-(2-naphthyl)-1*H*-1,2,3-triazol-4-yl]methylamino}-5-*O*-mycaminosyltylonolide (**1**), 20-deoxy-20-{*N*-methyl-*N*-[1-(6-quinolyl)-1*H*-1,2,3-triazol-4-yl]methylamino}-5-*O*-mycaminosyltylonolide (**2**), 20-deoxy-20-{*N*-methyl-*N*-[1-(3-quinolyl)-1*H*-1,2,3-triazol-4-yl]methylamino}-5-*O*-mycaminosyltylonolide (**3**), 20-deoxy-20-{*N*-methyl-*N*-[1-(3-quinolyl)-1*H*-1,2,3-triazol-4-yl]methylamino}-5-*O*-mycaminosyltylonolide (**4**), 20-deoxy-20-{*N*-benzyl-*N*-[1-(3-quinolyl)-1*H*-1,2,3-triazol-4-yl]methylamino}-5-*O*-mycaminosyltylonolide (**5**), 20-deoxy-20-{*N*-methyl-*N*-[1-(1-naphthyl)-1*H*-1,2,3-triazol-4-yl]methylamino}-5-*O*-mycaminosyltylonolide (**6**)), and 15-membered macrolides (3-*O*-Descladinosyl-3-*O*-[2-(2-pyridyl)acetyl]-6-*O*-(3-{2-[(3-carboxy-6-fluoro-1-cyclopropyl-1,4-dihydro-4-oxoquinolin-7-yl)piperazin-1-yl]}propanoyl)azithromycin (**7**), 3-*O*-Descladinosyl-3-*O*-[2-(2-pyridyl)acetyl]-6-*O*-(3-{2-[3-(3-carboxy-6-fluoro-8-methoxy-1-cyclopropyl-1,4-dihydro-4-oxoquinolin-7-yl)methylpiperazin-1-yl]}propanoyl)azithromycin (**8**)) (Figure 3 and Figure 4)—was performed using MacroModel under the Schrodinger Suite 2021-1 and with Maestro v. 12.7.156 as the interface. Chloroform was used as the solvent. The minimisations were first performed with charges from the force field (AMBER^*^, MM2), the cut-off was extended, the minimisation method was TNCG (Truncated Newton Conjugate Gradient), and the maximum number of iterations was set to 10,000, with the gradient convergence, and its threshold of 0.05. Conformational search torsional sampling was MCMM (Monte Carlo multiple minimum) with automatic setup during the calculation, and torsion sampling options were set to intermediate. The maximum number of steps was 10,000, with 100 steps per rotatable bond. The number of structures to be saved for each search was 100, energy window for saving structures was 21 kJ/mol, and the maximum atom deviation cut-off was 0.5 Å.

The constrained conformational search was performed on erythromycin A, erythromycin C, 3-*O*-Descladinosyl-3-*O*-[2-(2-pyridyl)acetyl]-6-*O*-(3-{2-[(3-carboxy-6-fluoro-1-cyclopropyl-1,4-dihydro-4-oxoquinolin-7-yl)piperazin-1-yl]}propanoyl)azithromycin (**7**), and 3-*O*-Descladinosyl-3-*O*-[2-(2-pyridyl)acetyl]-6-*O*-(3-{2-[3-(3-carboxy-6-fluoro-8-methoxy-1-cyclopropyl-1,4-dihydro-4-oxoquinolin-7-yl)methylpiperazin-1-yl]}propanoyl)azithromycin (**8**). In all cases, the conformational search was set using the global minimum energy structure from the unconstrained conformational search, with the following distance constraints: H4–H11 (2.5 ± 0.3 Å) and H5–H18 (2.5 ± 0.3 Å). 

#### 3.2.3. Molecular Docking Studies

Molecular docking studies with chain B of the protein entity of the 4V7U entry from the Protein Data Bank and previously prepared ligands (selected macrolide antibiotics) in MacroModel under Schrodinger 2021-1 and Maestro 12.7 were performed in Glide under Schrodinger 2021-2 and Maestro 12.8. To get better insight, the binding site of erythromycin A to *E. coli* from 4V7U entry (consisting of both amino acids and nucleotides) together with optimised selected macrolide antibiotics were also submitted to the Patchdock server, and then to Firedock for refinement. 

Before molecular docking with Glide [64,65], we firstly had to prepare the target for the process. In Maestro 12.8, we imported entry 4V7U, and removed all except chains of the proteins, and then used Protein Preparation Wizard for pre-processing, removing all protein chains except chain B (consisting of amino acids from the binding site) and optimization. The receptor grid generation process gave a grid file with the coordinates necessary for the molecular docking. The ligand (selected macrolide antibiotic) was then imported to perform ligand docking with the standard precision. 

Molecular docking studies were also performed using the Patchdock web server with the Patchdock molecular docking algorithm based on shape complementarity principles [66,67,68]. The input was set consisting of the pdb files of the receptor molecule and the ligand molecule. The pdb files of the ligand molecules consisted of folded-out structures obtained in the unconstrained conformational search for all investigated 14- and 15-membered macrolide antibiotics (erythromycin A, erythromycin B, erythromycin C, 3-*O*-Descladinosyl-3-*O*-[2-(2-pyridyl)acetyl]-6-*O*-(3-{2-[(3-carboxy-6-fluoro-1-cyclopropyl-1,4-dihydro-4-oxoquinolin-7-yl)piperazin-1-yl]}propanoyl)azithromycin (**7**), 3-*O*-Descladinosyl-3-*O*-[2-(2-pyridyl)acetyl]-6-*O*-(3-{2-[3-(3-carboxy-6-fluoro-8-methoxy-1-cyclopropyl-1,4-dihydro-4-oxoquinolin-7-yl)methylpiperazin-1-yl]}propanoyl)azithromycin (**8**)), and selected 16-membered macrolide antibiotics (rosamicin, tildipirosin, tylosin A, tylosin B, 20-deoxy-20-{*N*-methyl-*N*-[1-(2-naphthyl)-1*H*-1,2,3-triazol-4-yl]methylamino}-5-*O*-mycaminosyltylonolide (**1**), 20-deoxy-20-{*N*-methyl-*N*-[1-(6-quinolyl)-1*H*-1,2,3-triazol-4-yl]methylamino}-5-*O*-mycaminosyltylonolide (**2**), 20-deoxy-20-{*N*-methyl-*N*-[1-(3-quinolyl)-1*H*-1,2,3-triazol-4-yl]methylamino}-5-*O*-mycaminosyltylonolide (**3**), 20-deoxy-20-{*N*-methyl-*N*-[1-(3-quinolyl)-1*H*-1,2,3-triazol-4-yl]methylamino}-5-*O*-mycaminosyltylonolide (**4**), 20-deoxy-20-{*N*-benzyl-*N*-[1-(3-quinolyl)-1*H*-1,2,3-triazol-4-yl]methylamino}-5-*O*-mycaminosyltylonolide (**5**), 20-deoxy-20-{*N*-methyl-*N*-[1-(1-naphthyl)-1*H*-1,2,3-triazol-4-yl]methylamino}-5-*O*-mycaminosyltylonolide (**6**)). The output is a list of the potential complexes sorted by the shape complementarity principles. Ten of the best results from the docking analysis using the Patchdock algorithm were submitted to FireDock for refinement [69,70]. Visualisation of the results and the formation of 2D diagrams were performed using Discovery Studio 2021 Client (Dessault Systems Biovia Corp., San Diego, CA, USA).

#### 3.2.4. ADMET Studies

ADMET parameters of the selected macrolides were calculated with the help of QikProp v7.0 software running in normal mode (Schrödinger LLC. QikProp. Schrödinger, LLC; New York, NY, USA: 2021-4). QikProp also allows providing acceptable ranges for comparing the predicted properties of compounds with those of 95% of known drugs, allowing the estimation of drug-likeness properties. 

Additionally, the online platform ADMETLab 2.0 was used for accurate and comprehensive predictions of ADMET properties [71].

### 3.3. Bacterial Strains

For the determination of MICs (Minimum Inhibitory Concentrations) of selected macrolide antibiotics (clarithromycin, azithromycin, tylosin A and B), the following bacterial strains were used: wild-type wound isolates of *Escherichia coli*, *Staphylococcus aureus*, *Serratia marcescens*, and *Corynebacterium xerosis* (derived from chronic wounds), *Bacillus cereus* MRBG 4.21 (isolated for a domestic drain as in PMID: 12957932), *Pseudomonas aeruginosa* (PA01 biofilm producing isolate), *Staphylococcus epidermidis* (a wild-type skin isolate).

### 3.4. MIC Determination

MICs were determined using standard methods [72]. Inocula for broth dilution end-point determination of bacterial antimicrobial susceptibility were prepared as follows: single colonies of test bacteria from axenic agar cultures were inoculated into sterile, nutrient broth (10 mL) contained in 25 mL sterile plastic universals and incubated in a standard aerobic incubator at 37 °C for 24 h. Cultures were then adjusted to 10^5^ CFU mL^−1^ using a MacFarland standard. Stock samples of clarithromycin, azithromycin, tylosin A, and tylosin B were prepared in distilled water. Microtitre plates (96-well) were used for the tests (Becton Dickinson, Franklin Lakes, NJ, USA). A diluted overnight culture (100 μL) was delivered to each test well, except the first column of the plate containing 100 μL of the antibiotic solution in distilled water diluted by 100 μL of double-strength nutrient broth, and the last two columns of the plate, which contained 100 μL of single-strength broth and 100 μL of distilled water, respectively. Doubling dilutions was then carried out across the plate using a multi-channel pipette, changing the tips at each dilution step. Then, the incubation for 24 h was performed in a standard aerobic incubator at 37 °C. Growth was detected as turbidity, relative to an uninoculated well using a microtitre plate reader (Powerwave XS; BioTek, Bedfordshire, UK). The MIC was determined as the lowest concentration of antibiotic that inhibited growth. 

## 4. Conclusions

There is no universal pattern for the construction of the active macrolide antibiotic against Gram-negative bacteria like Escherichia coli. The mining for a macrolide active against Gram-negative bacteria depends on the size of the lactone ring in the macrolide. Fifteen-membered macrolides with W values of 0.0762 and 0.0767 Ry or quite similar will have activities against Gram-negative bacteria. In the case of 16-membered macrolides, those with W values of 0.0841, 0.0806, 0.0765, 0.0739, 0.0731, 0.0909 Ry or similar will have significant activities against Gram-negative bacteria. The proper value of electron-ion interaction potential together with the interactions with the amino acids from or near the binding site, and no or one hydrogen bonding interaction with nucleotides, provide the key to the active macrolide against E. coli. According to ADMET study, only rosaramicin (VRF = 1) can be considered as a good oral drug candidate. Considering values for VRT, beside rosaramicin (VRT = 0), the drugs already known such as erythromycin A, B, and C, and tylosin A and B, are oral drugs (VRT = 1). Lipinski’s rule of five and in silico ADMET analysis with commercial software should be taken with a reserve in case of macrolides, and models and rules should be developed separately due to their chameleon-like conformational flexibility and frequent interaction with cellular membrane transporters. Toxicity profiles of the investigated macrolides show that they can be used with the caution similarly demonstrated to other commercially available medicines, such as azithromycin and artemisinin. Complete NMR analysis was performed on tylosin A in deuterated chloroform and the obtained data were used to investigate the conformation of the macrolide in the solution. 

## Figures and Tables

**Table 1:** The chemical shifts, coupling constants, and HMBC connectivities for tylosin A in CDCl_3_ at 20 °C on Bruker Avance 400 MHz and 500 MHz NMR spectrometers (Karlsruhe, Germany).

Position	Multiplicity	^1^H (ppm)	J_HH_ (Hz)	^13^C (ppm)	HMBC Connectivities (^13^C→^1^H)
1	-	-	-	174.0	H_2_2, H3, H15
2	m	1.99 2.57	-	39.4	H_3_18
3	m	3.81	-	69.7	-
4	m	1.71	-	40.7	-
5	m	3.53	-	81.8	H3, H_3_18
6	m	1.65	-	30.6	-
7	bs m	1.50 1.96	-	32.2	-
8	m	2.57	-	45.0	-
9	-	-	-	>203.4	H10, H11, H_3_21
10	dd	6.33	14.85	118.4	-
11	dd	7.33	15.05	148.2	H13, H_3_22
12	-	-	-	135.0	H10, H11, H14, H_3_22
13	dd	5.92	9.25	142.3	H11, H14, H_3_22, H_2_23
14	d	2.98	6.2	45.0	H13, H15, H_2_16, H_2_23
15	m	4.98	-	70.0	H2, H_2_16, H_3_17, H_2_23
16	m m	1.63 1.88	-	25.4	H15, H14, H_3_17
17	s	0.94	-	9.8	H15, H_2_16, H_3_22
18	s	1.00	-	9.0	H_2_2, H3, H5
19	m m	2.43 2.90	- -	43.7	-
20	s	9.68	-	203.4	H_2_19, H_3_21
21	s	1.23	-	17.8	H_2_7, H20
22	s	1.81	-	13.1	H_2_14, H_3_17, H23
23	dd m	3.76 4.00	5.3 -	69.1	H13
1′	s	4.40	-	103.3	-
2′	m	3.82	-	75.2	-
3′	m	3.37	-	70.6	H_3_6′
4′	m	3.35	-	72.6	-
7′8′	m	2.80	-	40.9	-
5′	m	3.37	-	73.3	-
6′	s	1.27	-	19.0	-
1″	s	5.13	-	95.3	-
2″	m m	2.05 2.51	-	40.7	H_3_7”
3″	-	-	-	70.6	H_3_7”
4″	d	3.19	9.20	76.4	-
5″	m	3.76	-	66.8	H1”, H_3_7”
6″	s	1.27	-	18.3	H4”
7″	s	1.26	-	25.7	H_3_6”
1‴	d	4.57	7.65	103.3	H_2_23, H2‴, H_3_OCH_3_2‴, H5‴, H6‴
2‴	dd	3.03	6.35 2.50	81.8	H_3_OCH_3_2‴, H_3_OCH_3_3‴, 5‴
OCH_3_2‴	s	3.49	-	59.8	H2‴, H_3_OCH_3_3‴, H4‴, H5‴
3‴	m	3.69	-	79.8	H1‴, H_3_OCH_3_3‴, H4‴
OCH_3_3‴	s	3.62	-	61.8	H5‴, H_3_OCH_3_2‴
4‴	m	3.37	-	70.6	H2‴, H_3_OCH_3_2‴, H5‴, H_3_6‴
5‴	m	3.76	-	70.0	-
6‴	s	1.27	-	17.4	H4

**Table 2:** Set of selected macrolides with calculated Z^*^ and W values.

Compound	Formula	Z^*^ (Ry)	W (Ry)
Erythromycin A	C_37_H_67_NO_13_	2.5254	0.0927
Erythromycin B	C_37_H_67_NO_12_	2.4957	0.0949
Erythromycin C	C_36_H_65_NO_13_	2.5392	0.0913
Tylosin A	C_46_H_77_NO_17_	2.61	0.0812
Tylosin B	C_39_H_65_NO_14_	2.605	0.0821
Tildipirosin	C_41_H_71_N_3_O_8_	2.4227	0.0962
Rosaramicin	C_31_H_51_NO_9_	2.5434	0.0909
20-deoxy-20-{*N*-methyl-*N*-[1-(2-naphthyl)-1*H*-1,2,3-triazol-4-yl]methylamino}-5-*O*-mycaminosyltylonolide (**1**)	C_45_H_65_N_5_O_9_	2.6129	0.0806
20-deoxy-20-{*N*-methyl-*N*-[1-(6-quinolyl)-1*H*-1,2,3-triazol-4-yl]methylamino}-5-*O*-mycaminosyltylonolide (**2**)	C_44_H_64_N_6_O_9_	2.6341	0.0765
20-deoxy-20-{*N*-methyl-*N*-[1-(3-quinolyl)-1*H*-1,2,3-triazol-4-yl]methylamino}-5-*O*-mycaminosyltylonolide (**3**)	C_43_H_62_N_6_O_9_	2.65	0.0731
20-deoxy-20-{*N*-methyl-*N*-[1-(3-quinolyl)-1*H*-1,2,3-triazol-4-yl]methylamino}-5-*O*-mycaminosyltylonolide (**4**)	C_44_H_64_N_6_O_9_	2.6341	0.0765
20-deoxy-20-{*N*-benzyl-*N*-[1-(3-quinolyl)-1*H*-1,2,3-triazol-4-yl]methylamino}-5-*O*-mycaminosyltylonolide (**5**)	C_50_H_68_N_6_O_9_	2.6467	0.0739
20-deoxy-20-{*N*-methyl-*N*-[1-(1-naphthyl)-1*H*-1,2,3-triazol-4-yl]methylamino}-5-*O*-mycaminosyltylonolide (**6**)	C_45_H_65_N_5_O_9_	2.6129	0.0806
3-*O*-Descladinosyl-3-*O*-[2-(2-pyridyl)acetyl]-6-*O*-(3-{2-[(3-carboxy-6-fluoro-1-cyclopropyl-1,4-dihydro-4-oxoquinolin-7-yl)piperazin-1-yl]}propanoyl)azithromycin (**7**)	C_57_H_84_FN_6_O_14_	2.6358	0.0762
3-*O*-Descladinosyl-3-*O*-[2-(2-pyridyl)acetyl]-6-*O*-(3-{2-[3-(3-carboxy-6-fluoro-8-methoxy-1-cyclopropyl-1,4-dihydro-4-oxoquinolin-7-yl)methylpiperazin-1-yl]}propanoyl)azithromycin (**8**)	C_59_H_88_FN_6_O_15_	2.633	0.0767

**Table 3:** Docking scores of selected macrolides to the narrow region around amino acids Lys 90 and Arg 92 of protein chain B from 70S *E. coli* ribosome.

Macrolide	Docking Score	E_best model_	E	E_int_
**1**	−2.607	−21.25	−21.86	7.30
**2**	−2.336	−21.31	−21.60	6.12
**3**	−2.884	−24.22	−23.56	6.44
**4**	−2.599	−27.69	−25.78	3.85
**5**	−2.202	−16.24	−17.60	6.34
**6**	−2.818	−23.20	−24.06	7.63
**7**	−0.801	−10.75	−14.46	7.47
**8**	−2.018	−17.66	−20.25	5.96
tildipirosin	−1.708	−13.33	−15.73	5.24

**Table 4:** Calculated absorption, distribution, metabolism, elimination, and toxicity (ADMET) parameters of the compounds.

Comp	MW	RB	DM	MV	DHB	AHB	PSA	log P	log S	PCaco	PM	HOA (%)	VRF	VRT	hergK+
Erythr A	733.9	12	5.4	1976.1	4	19.1	132.6	2.89	−2.85	157	9	57	2	1	−4.89
Erythr B	717.9	11	5.2	1916.2	3	18.4	119.6	2.24	−2.47	197	8	55	2	1	−4.51
Erythr C	719.9	12	7.1	1932.1	5	19.1	143.7	1.75	−2.78	98	10	34	3	1	−4.91
Tylosin A	916.1	32	8.6	1132.7	4	28.1	199.2	1.47	0.13	26	11	35	2	1	−5.88
Tylosin B	771.9	29	5.6	990.0	3	24.0	163.2	0.78	0.54	55	10	37	2	1	−5.34
Tildip	734.1	26	8.1	1038.1	2	17.5	114.3	3.13	−0.4	7	10	35	2	2	−7.10
Rosaramicin	581.7	21	10.7	769.1	1	15.8	135.1	1.46	0.47	89	6	57	1	0	−4.20
**1**	820.1	28	5.9	1169.1	3	19.2	165.3	3.67	−2.96	4	11	34	2	2	−8.65
**2**	821.0	28	10.8	2289.4	3	20.2	177.6	2.45	−1.42	2	12	23	2	2	−8.11
**3**	806.9	28	10.5	1109.2	4	19.7	187.4	2.81	−1.87	2	12	24	2	2	−7.95
**4**	821.1	28	6.0	1164.6	3	20.2	177.7	3.00	−2.51	2	12	Low	2	2	−8.51
**5**	897.1	30	9.9	1232.3	3	20.2	164.5	4.82	−3.03	7	13	45	2	2	−9.36
**6**	820.1	28	10.2	1119.1	3	19.2	159.5	3.72	−2.14	8	11	39	2	2	−8.08
**7**	1095.3	15	9.9	2825.2	3	25.1	205.3	1.99	−4.25	0	10	2	2	2	−6.95
**8**	1139.4	16	7.4	2839.9	3	25.8	195.3	1.68	−2.87	0	11	0	2	2	−6.04

**MW**: Molecular weight; **RB**: Number of rotatable bonds; **DM**: computed dipole moment; **MV**: total solvent-accessible volume; **DHB**: estimated number of hydrogen-bond donors; **AHB**: estimated number of hydrogen-bond acceptors; **PSA**: van der Waals surface area of polar nitrogen and oxygen atoms and carbonyl carbon atoms; **logP**: predicted octanol/water partition coefficient; **log S**: predicted aqueous solubility; **PCaco**: predicted apparent Caco-2 cell permeability; **PM**: number of likely metabolic reactions; **% HOA**: predicted human oral absorption percentage; **VRF**: number of violations of Lipinski’s rule of five (the rules are as follows: MW < 500, log P < 5, DHB ≤ 5, AHB ≤ 10, positive PSA value); **VRT**: number of violations of Jorgensen’s rule of three (the rules are as follows: log S > −5.7, PCaco > 22 nm/s, PM < 7); **hergK+**: Human Enter-a-go-go Related Gene (concern below −5); **Erythr A**: erythromycin A; **Erythr B**: erythromycin B; **Erythr C**: erythromycin C; **Tildip**: tildipirosin.

**Table 5:** Toxicity profiles of selected macrolides and control compounds (azithromycin and artemisinin) in use as medicines.

Descriptors		Toxicity										Tox21 Rules							
	Compounds	hERG blockers	H-HT	DILI	Respiratory toxicity	AMES toxicity	FDAMDD	Skin Sensitization	Carcinogenicity	Eye Irritation	Eye Corrosion	NR-AR	NR-aromatase	NR-AR-LBD	NR-ER	NR-ER-LBD	SR-ARE	SR-MMP	SR-P53
Azithromycin		-	+++	+++	+++	---	---	---	---	---	---	---	---	---	-	---	---	---	---
Artemisinin		++	+++	+++	---	++	---	+	++	+	+	---	---	++	---	+	---	---	+++
Erythromycin A		---	++	+++	---	---	---	---	---	---	---	---	---	---	+	---	---	-	+
Erythromycin B		---	++	+++	+	---	---	---	---	---	---	---	---	---	+	---	---	---	---
Erythromycin C		---	++	+++	---	---	---	---	---	---	---	-	---	---	+	---	---	---	---
Tildipirosin		-	-	---	+++	---	-	++	---	---	---	---	---	---	+	---	+	---	---
Tylosin A		+	-	+	+++	---	---	++	---	---	---	---	-	---	+	-	+	-	+
Tylosin B		++	-	---	+++	---	---	++	---	---	---	-	---	---	+	+	+	---	---
Rosaramicin		---	-	-	+++	---	-	---	++	---	---	---	---	---	+	++	---	---	---
**1**		+	+	+++	+++	---	+	---	---	---	---	---	---	---	+	---	+	---	---
**2**		+	+	+++	+++	---	+	---	---	---	---	---	---	---	+	---	+	---	---
**3**		+	++	+++	+++	---	+	---	---	---	---	---	---	---	+	---	+	---	---
**4**		-	-	+++	+++	---	+	---	---	---	---	---	---	---	+	---	+	---	---
**5**		++	++	+++	+++	---	+	---	---	---	---	---	---	---	+	---	+	-	---
**6**		+	+	++	+++	---	-	---	---	---	---	-	---	---	+	---	+	---	---
**7**		+	+++	+++	++	---	+	---	---	---	---	++	---	---	+	---	+	---	---
**8**		+	+++	+++	+++	---	+	---	---	---	---	++	---	---	+	---	+	---	---

**Table 6:** Minimum inhibitory concentrations (MICs) expressed in µg/mL for tylosin A and tylosin B compared to clarithromycin and azithromycin.

Bacterial Strains	MIC (µg/mL)			
	Clarithromycin	Azithromycin	Tylosin A	Tylosin B
*Escherichia coli*	12	2	125	31.25
*Bacillus cereus* MRBG 4.21	0.2	4	0.98	0.98
*Staphylococcus aureus*	0.2	8	31.25	>500
*Pseudomonas aeruginosa* PA01	62.5	15.6	250	62.5
*Staphylococcus epidermidis*	250	31.2	250	62.5
*Serratia marcescens*	>500	>500	500	250
*Corynebacterium xerosis*	>500	250	500	250

**Table 7:** MICs of selected macrolides.

Bacterial Strains	MIC (µg/mL)										Reference
	1	2	3	4	5	6	7	8	Tild.	Ros.	
*E. coli* NIHJ JC-2	8	16	8	4	16	16	NA	NA	8	ND	[26]
*E. coli* EC14	NA	NA	NA	NA	NA	NA	NA	NA	NA	16	[25]
*E. coli* BW25113	NA	NA	NA	NA	NA	NA	4	2	NA	NA	[61]

## Data Availability

All data necessary for understanding of the research are present in the manuscript. Additional data related to molecular modelling and NMR analyses are available as Supplementary Material.

## Supplementary Materials

The following supporting information can be downloaded at: https://www.mdpi.com/article/10.3390/molecules27217280/s1. Table S1: 500 MHz ROESY interactions for tylosin A in CDCl_3_. TMS was used as an internal standard. (L = large, M = medium, S = small, V = very small). Table S2: Distances obtained for tylosin A in chloroform using 2D ROESY NMR and molecular modelling; Symbols: L = Large, M = Medium, S = Small, V = Very small. Figure S1: (a) Part of ^1^H NMR spectrum of tylosin A in CDCl_3_ from 0–2.3 ppm, (b) part of ^1^H NMR spectrum of tylosin A in CDCl_3_ from 2.3–5.3 ppm, (c) part of ^1^H NMR spectrum of tylosin A in CDCl_3_ from 5.6–9.8 ppm. Figure S2: ^13^C NMR spectrum of tylosin A in CDCl_3_. Figure S3: (a) ^13^C DEPT 135 NMR spectrum of tylosin A in CDCl_3_, (b) enlarged ^13^C DEPT 135 NMR spectrum of tylosin A in CDCl_3_ from 55–90 ppm. Figure S4: 2D DQFCOSY NMR spectrum of tylosin A in CDCl_3_. Figure S5: 2D HMBC NMR spectrum of tylosin A in CDCl_3_ showing interactions belonging to tylosin A, but also the additional signals tentatively assigned to the degradant tylosin aldol. Circled peaks are not real HMBC cross peaks of tylosin A or do not belong to this compound. Figure S6: 2D HMQC NMR of tylosin A in CDCl_3_ showing interactions belonging to tylosin A, but also the additional signals tentatively assigned to the degradant tylosin aldol. Figure S7: 2D ROESY NMR spectrum of tylosin A in CDCl_3_. Figure S8: Interactions between (**1**) (pink) and the amino acids from chain B from *E. coli* ribosome. Figure S9: Interactions between (**2**) (pink) and the amino acids from chain B from *E. coli* ribosome. Figure S10: Interactions between (**3**) (pink) and the amino acids from chain B from *E. coli* ribosome. Figure S11: Interactions between (**4**) (pink) and the amino acids from chain B from *E. coli* ribosome. Figure S12: Interactions between (**5**) (pink) and the amino acids from chain B from *E. coli* ribosome. Figure S13: Interactions between (**6**) (pink) and the amino acids from chain B from *E. coli* ribosome. Figure S14: Interactions between (**7**) (pink) and the amino acids from chain B from *E. coli* ribosome. Figure S15: Interactions between (**8**) (pink) and the amino acids from chain B from *E. coli* ribosome. Figure S16: Interactions between tildipirosin (pink) and the amino acids from chain B from *E. coli* ribosome. Figure S17: Interactions between (**3**) (red) and the nucleotides from the binding site from *E. coli* ribosome. Figure S18: Interactions between (**5**) (red) and the nucleotides from the binding site from *E. coli* ribosome. Figure S19: Interactions between (**6**) (red) and the nucleotides from the binding site from *E. coli* ribosome. Figure S20: Interactions between (**2**) (red) and the nucleotides from the binding site from *E. coli* ribosome. Figure S21: Interactions between (**4**) (red) and the nucleotides from the binding site from *E. coli* ribosome. Figure S22: Interactions between (**1**) (red) and the nucleotides from the binding site from *E. coli* ribosome. Figure S23: Interactions between rosaramicin (red) and the nucleotides from the binding site from *E. coli* ribosome. Figure S24: Interactions between (**8**) (red) and the nucleotides from the binding site from *E. coli* ribosome. Figure S25: Interactions between (**7**) (red) and the nucleotides from the binding site from *E. coli* ribosome. Figure S26: Interactions between erythromycin A (red) and the nucleotides from the binding site from *E. coli* ribosome. Figure S27: Interactions between erythromycin B (red) and the nucleotides from the binding site from *E. coli* ribosome. Figure S28: Interactions between erythromycin C (red) and the nucleotides from the binding site from *E. coli* ribosome. Figure S29: Interactions between tylosin A (red) and the nucleotides from the binding site from *E. coli* ribosome. Figure S30: Interactions between tylosin B (red) and the nucleotides from the binding site from *E. coli* ribosome.
