# Supplementary material for: Computational Studies on Selected Macrolides Active against Escherichia coli Combined with the NMR Study of Tylosin A in Deuterated Chloroform
Source: Molecules. 2022 Oct 26;27(21):7280. doi: 10.3390/molecules27217280 (PMC9654277; doi:10.3390/molecules27217280)
Supplement: Supplementary file 1 [file molecules-27-07280-s001.zip › molecules-1913692-supplementary.pdf]

	2s	2r	3	4	5	6	7s	7r	8	10	11	13	14	15	16s	16r	17	18	19s	19r	20	21	22	23s	23r	1'	2'	3'	4'	5'	6'	7'	1"	2"s	2"r	4"	5"	6"	7"	1‴	2‴	2c	3‴	4‴	5‴	6‴
2s	●																																													
2r		●																																												
3			●																																											
4				●														M																												
5					●																																									
6						●													V																											
7s							●																																							
7r								●																																						
8							S	S	●													S																								
10										●	V												V																							
11										V	●	L										V																								
13											L	●																																		
14													●		S								M	L	V																					
15												V		●			V																													
16s													S		●		S																													
16r																●																														
17														V			●																													
18																		●																												
19s								M											●																											
19r							S	S												●																										
20																					●																									
21							V	V														●																								
22																							●																							
23s																								●																						
23r																									●																					
1'																										●																				
2'																											●																			
3'																												●																		
4'																													●		M															
5'																														●																
6'																															●															
7'																																●														
1"																																	●					S								
2"s																																		●												
2"r																																			●											
4"																																				●		M								
5"																																				S	●									
6"																																				M		●								
7"																																							●							
1‴																																								●			V			
2‴																																								M	●					
2c																																									S	●	S			
3‴																																											●			
4‴																																								S				●		
5‴																																													●	
6‴																																								S	V					●
	2s	2r	3	4	5	6	7s	7r	8	10	11	13	14	15	16s	16r	17	18	19s	19r	20	21	22	23s	23r	1'	2'	3'	4'	5'	6'	7'	1"	2"s	2"r	4"	5"	6"	7"	1‴	2‴	2c	3‴	4‴	5‴	6‴

Table S1: 500 MHz ROESY interactions for tylosin A in $CDCl_3$. TMS was used as an internal standard. (L=large, M=medium, S=small, V=very small).

Contact	NMR spectrum (label and tolerance level of distances in Å)	Modelling (distances in Å)	
		MM2	AMBER*
4-18	M (2.3±0.5)	2.13	2.13
6-19s	V (3.5±1.5)	2.52	3.02
8-7s	S (2.5±1.0)	2.49	2.55
8-7r	S (2.5±1.0)	3.05	3.05
8-21	S (2.5±1.0)	2.15	2.12
10-11	V (3.5±1.5)	3.10	3.09
10-22	V (3.5±1.5)	5.98	2.27
11-10	V (3.5±1.5)	3.10	3.09
11-13	L (2.0±0.4)	3.39	2.38
11-21	V (3.5±1.5)	5.05	5.10
13-11	L (2.0±0.4)	3.39	2.38
14-16s	S (2.5±1.0)	3.01	3.84
14-22	M (2.3±0.5)	4.05	2.61
14-23r	V (3.5±1.5)	3.02	2.52
14-23s	L (2.0±0.4)	2.42	2.34
15-13	V (3.5±1.5)	2.95	3.77
15-17	V (3.5±1.5)	2.63	2.75
16s-17	S (2.5±1.0)	2.16	2.15
16s-14	S (2.5±1.0)	3.01	3.84
17-15	V (3.5±1.5)	2.63	2.75
19r-7s	S (2.5±1.0)	2.98	2.34
19s-7r	S (2.5±1.0)	4.35	3.12
21-7s	V (3.5±1.5)	2.55	2.54
21-7r	V (3.5±1.5)	2.68	2.86
4'-6'	M (2.3±0.5)	2.61	2.51
1''-6''	S (2.5±1.0)	4.40	4.43
4''-6''	M (2.3±0.5)	2.41	2.53
5¨-4¨	S (2.5±1.0)	3.06	2.51
6''-4''	M (2.3±0.5)	2.41	2.53
1'''-3'''	V (3.5±1.5)	3.89	2.45
2'''-1'''	M (2.3±0.5)	3.08	2.47
2'''OCH_3-1'''	S (2.5±1.0)	2.67	3.99
2'''OCH_3-3'''	S (2.5±1.0)	3.86	4.46
4'''-1'''	S (2.5±1.0)	2.49	3.93
6'''-1'''	S (2.5±1.0)	4.40	4.43
6'''-2'''	V (3.5±1.5)	2.22	4.07

Table S2: Distances obtained for tylosin A in chloroform using 2D ROESY NMR and molecular modelling; Symbols: L=Large, M=Medium, S=Small, V=Very small.

(a)

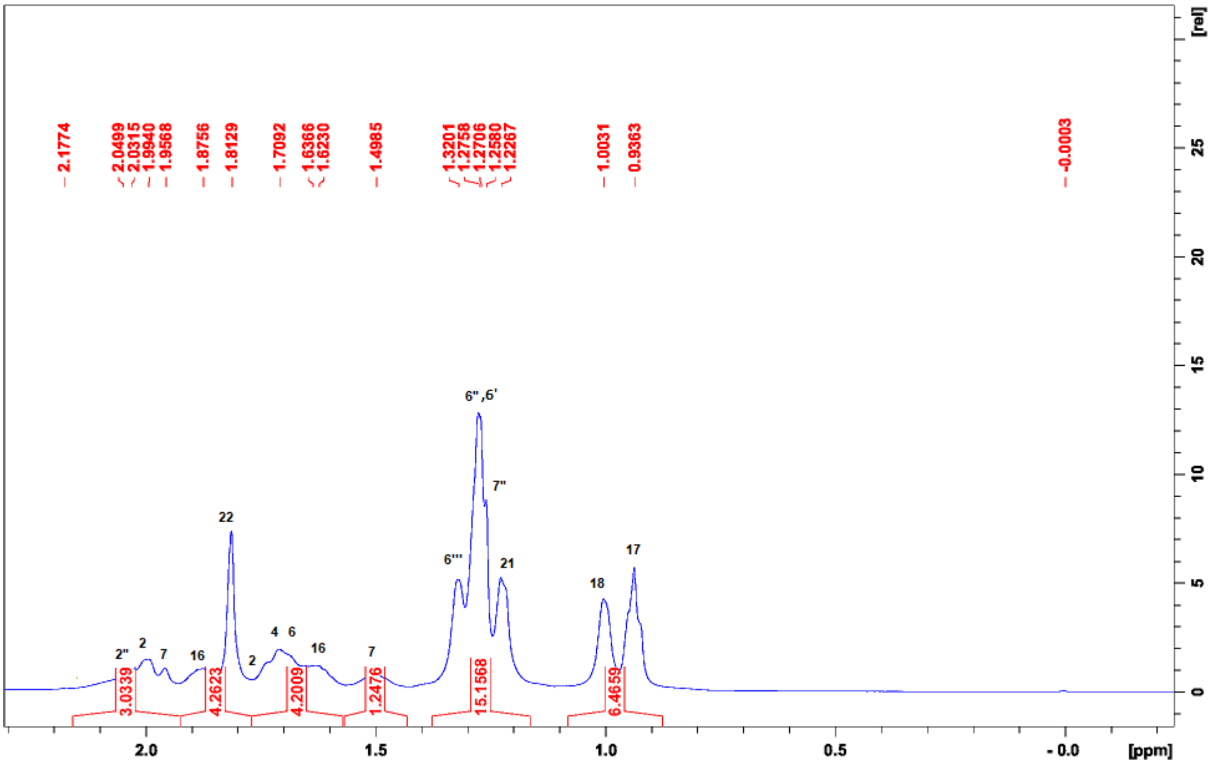

2.1774
2.0499
2.0315
1.9940
1.9568
1.8756
1.8129
1.7092
1.6366
1.6230
1.4985
1.3201
1.2758
1.2706
1.2580
1.2267
1.0031
0.9363
-0.0003
6",6'
7"
6'''
21
22
18
17
2"
2
7
16
2
4
6
16
7
3.0339
4.2623
4.2009
1.2476
15.1568
6.4659
[rel]
25
20
15
10
5
0
2.0
1.5
1.0
0.5
- 0.0
[ppm]

(b)

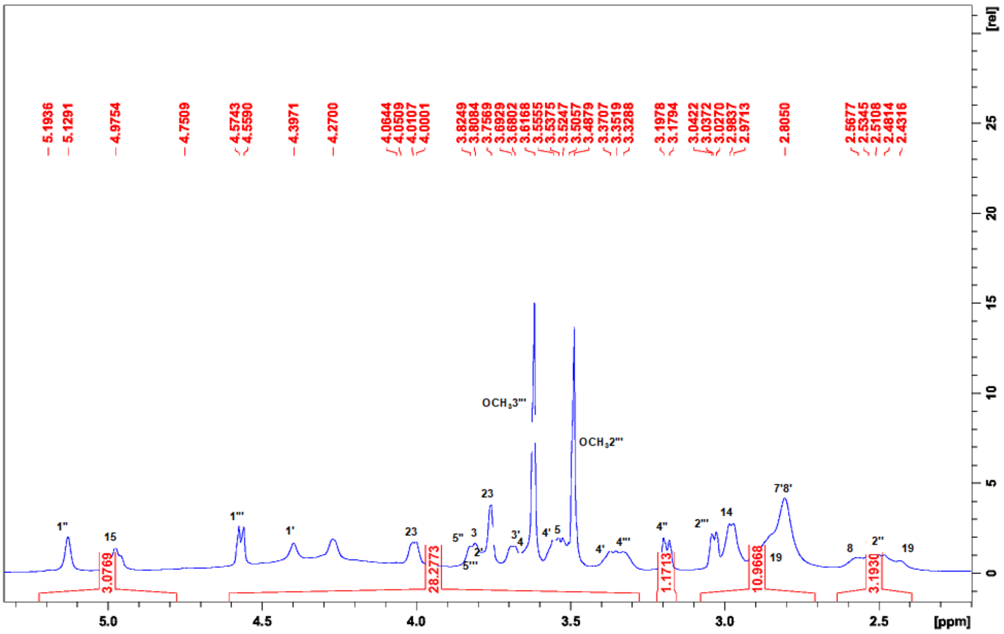

5.1936
5.1291
4.9754
4.7509
4.5743
4.5590
4.3971
4.2700
4.0644
4.0509
4.0107
4.0001
3.8249
3.8084
3.7569
3.6929
3.6802
3.6168
3.5555
3.5375
3.5247
3.5057
3.4879
3.3707
3.3519
3.3288
3.1978
3.1794
3.0422
3.0372
3.0270
2.9837
2.9713
2.8050
2.5677
2.5345
2.5108
2.4814
2.4316
OCH$_3$3'''
OCH$_3$2'''
7'8'
1"
15
1'''
1'
23
23
5"
3
2'
5'''
3'
4
4'
5
4'
4'''
4"
2'''
14
19
8
2"
19
3.0769
28.2273
1.1713
10.9668
3.1930
[rel]
25
20
15
10
5
0
5.0
4.5
4.0
3.5
3.0
2.5
[ppm]

(c)

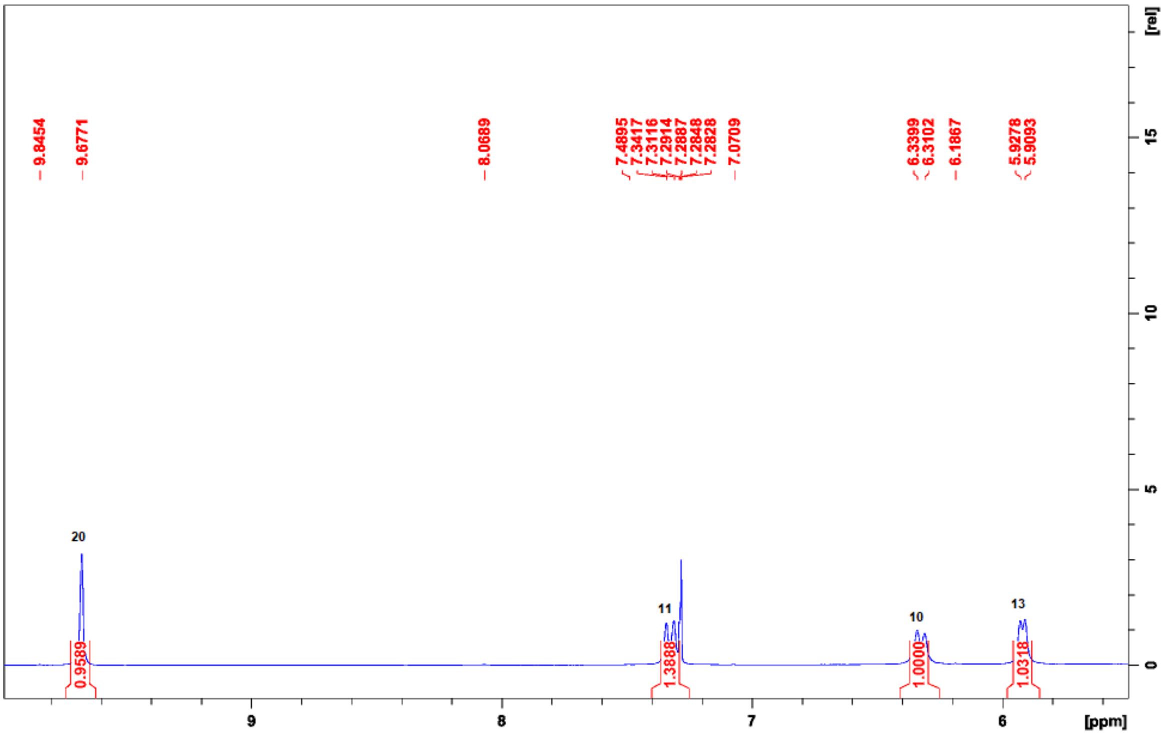

Figure S1: (a) Part of ^{1}H NMR spectrum of tylosin A in $CDCl_3$ from 0-2.3 ppm, (b) part of ^{1}H NMR spectrum of tylosin A in $CDCl_3$ from 2.3-5.3 ppm, (c) part of ^{1}H NMR spectrum of tylosin A in $CDCl_3$ from 5.6-9.8 ppm.

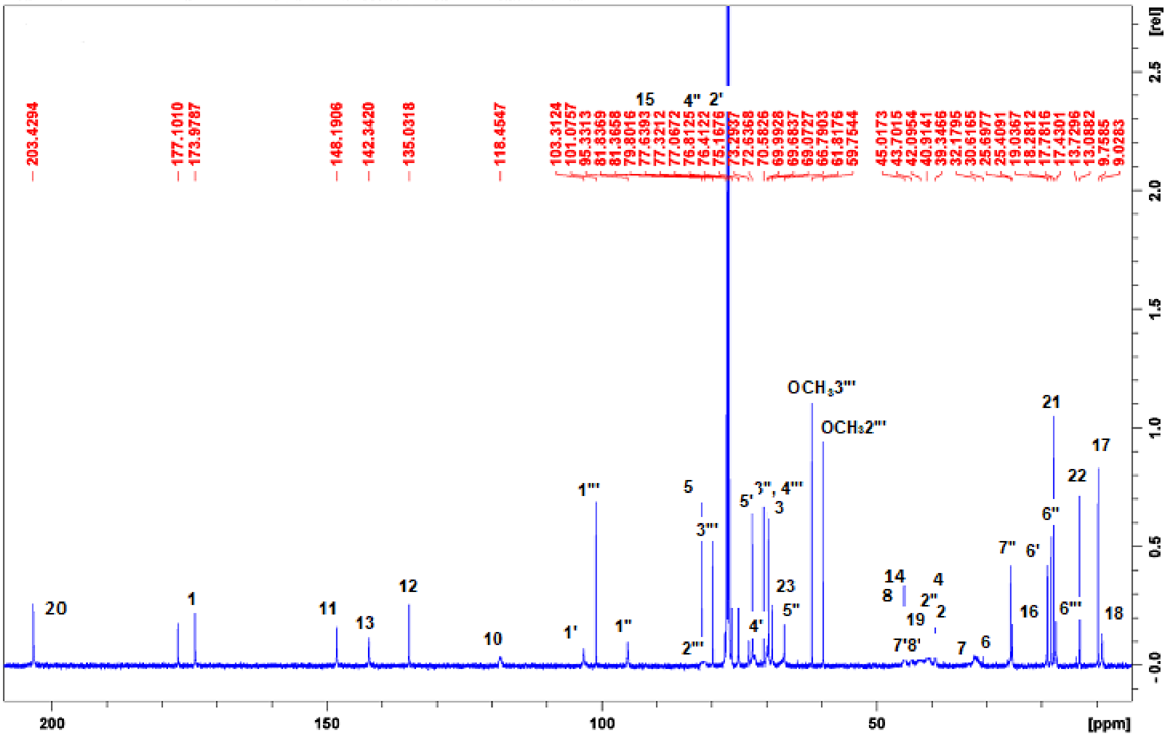

Figure S2: ^{13}C NMR spectrum of tylosin A in $CDCl_3$.

(a)

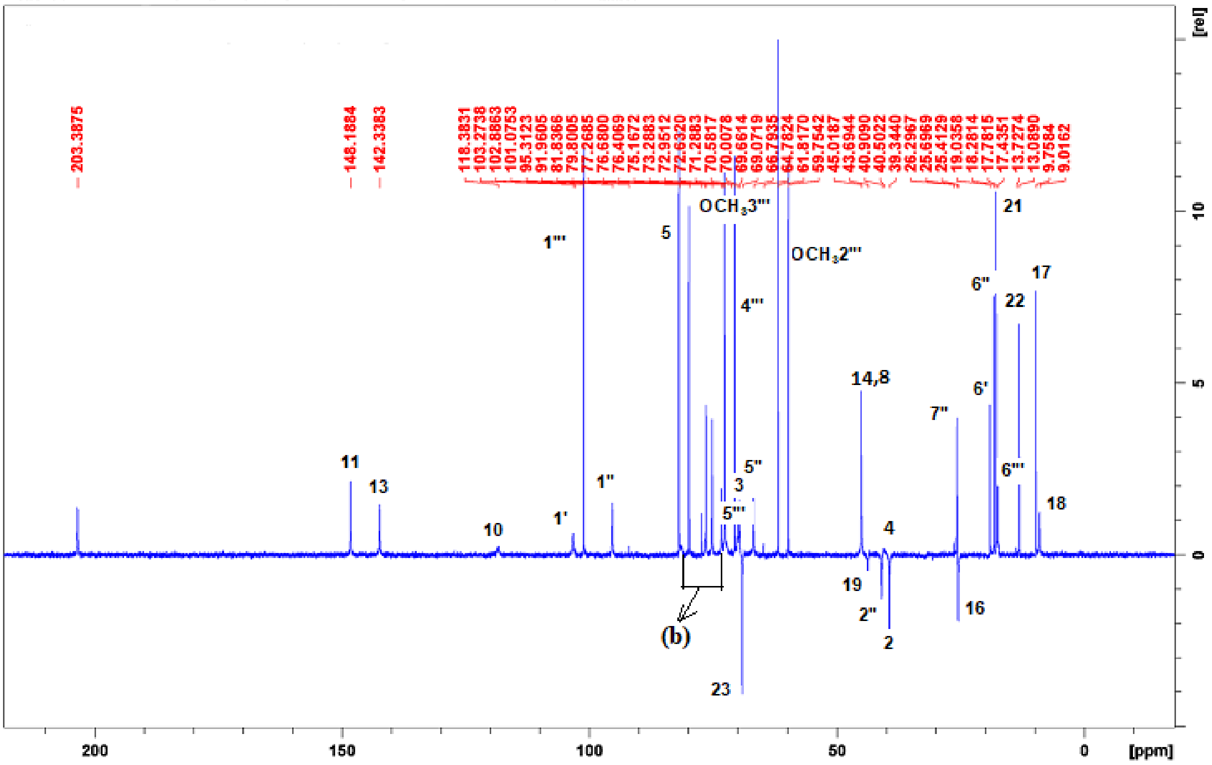

(b)

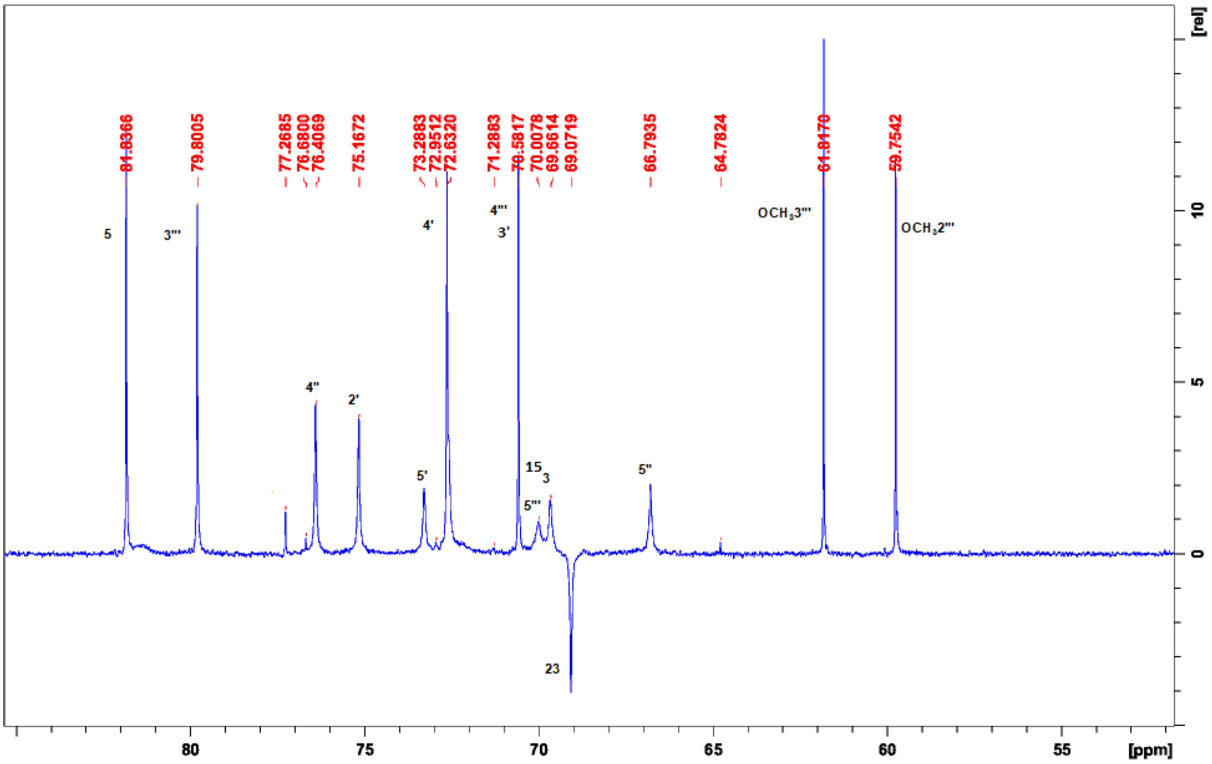

Figure S3: (a) ^{13}C DEPT 135 NMR spectrum of tylosin A in $CDCl_3$, (b) enlarged ^{13}C DEPT 135 NMR spectrum of tylosin A in $CDCl_3$ from 55-90 ppm.

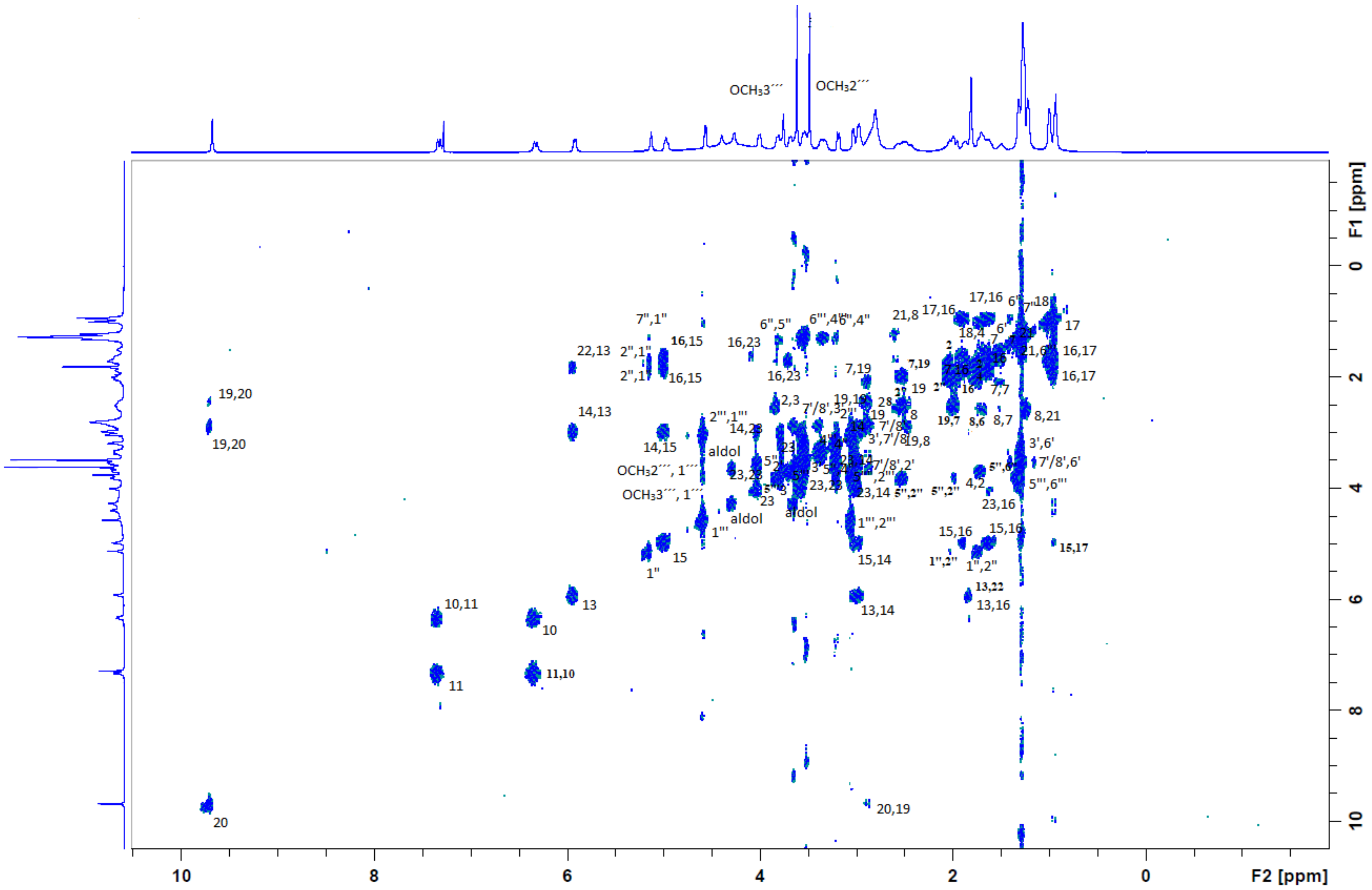

Figure S4: 2D DQFCOSY NMR spectrum of tylosin A in $CDCl_3$.

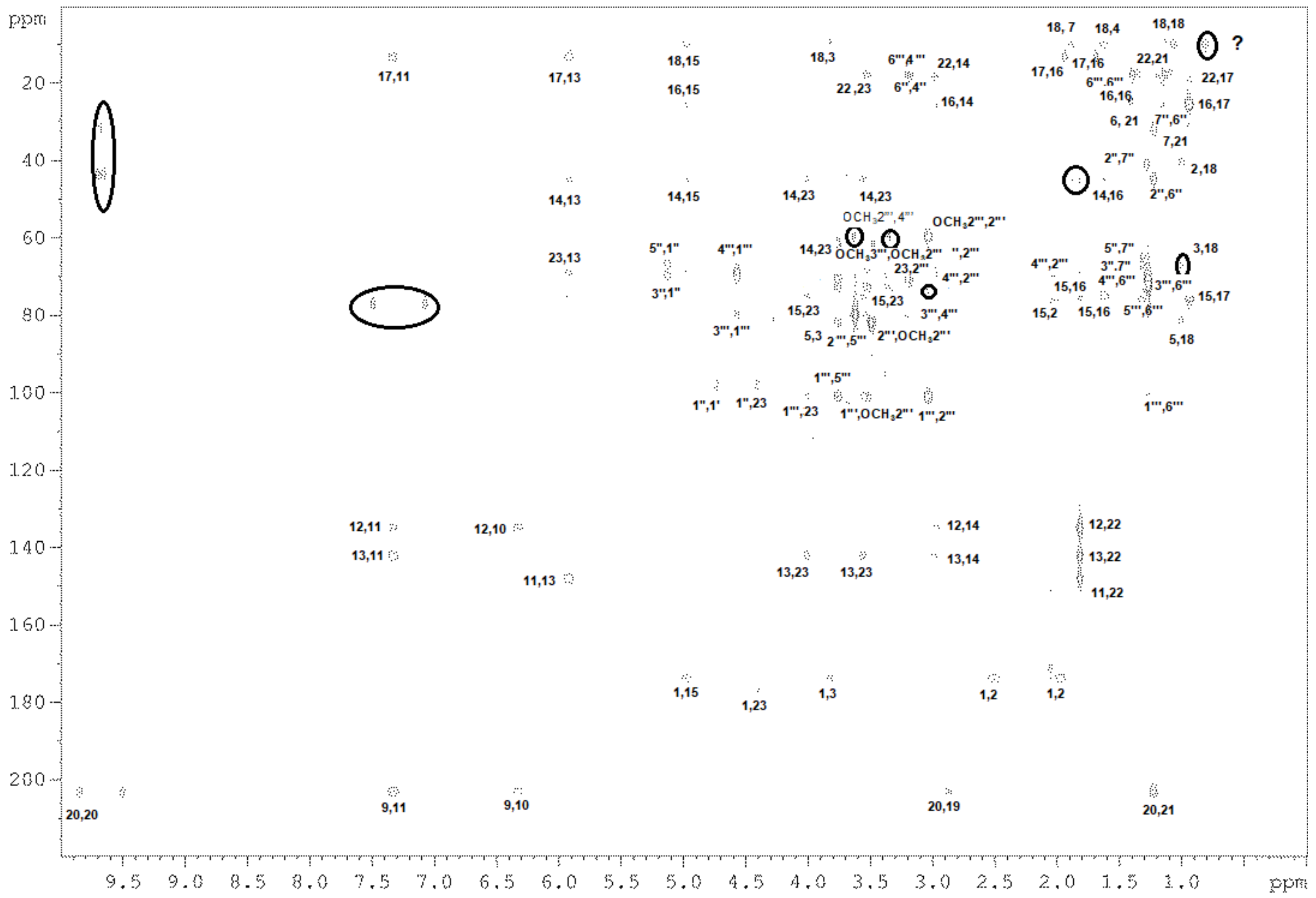

Figure S5: 2D HMBC NMR spectrum of tylosin A in $CDCl_3$ showing interactions belonging to tylosin A, but also the additional signals tentatively assigned to the degradant tylosin aldol. Circled peaks are not real HMBC cross peaks of tylosin A or do not belong to this compound.

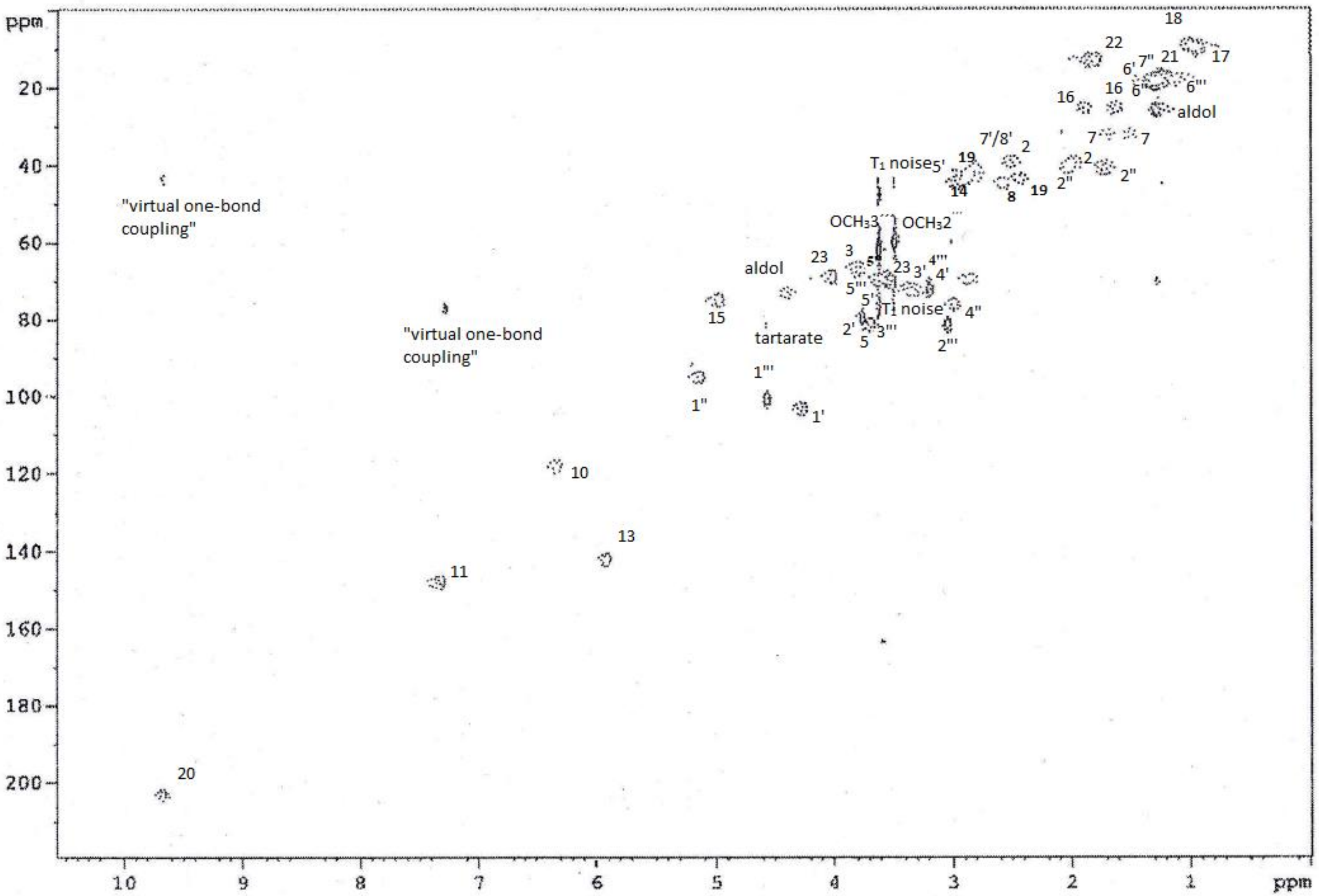

Figure S6: 2D HMQC NMR of tylosin A in $CDCl_3$ showing interactions belonging to tylosin A, but also the additional signals tentatively assigned to the degradant tylosin aldol.

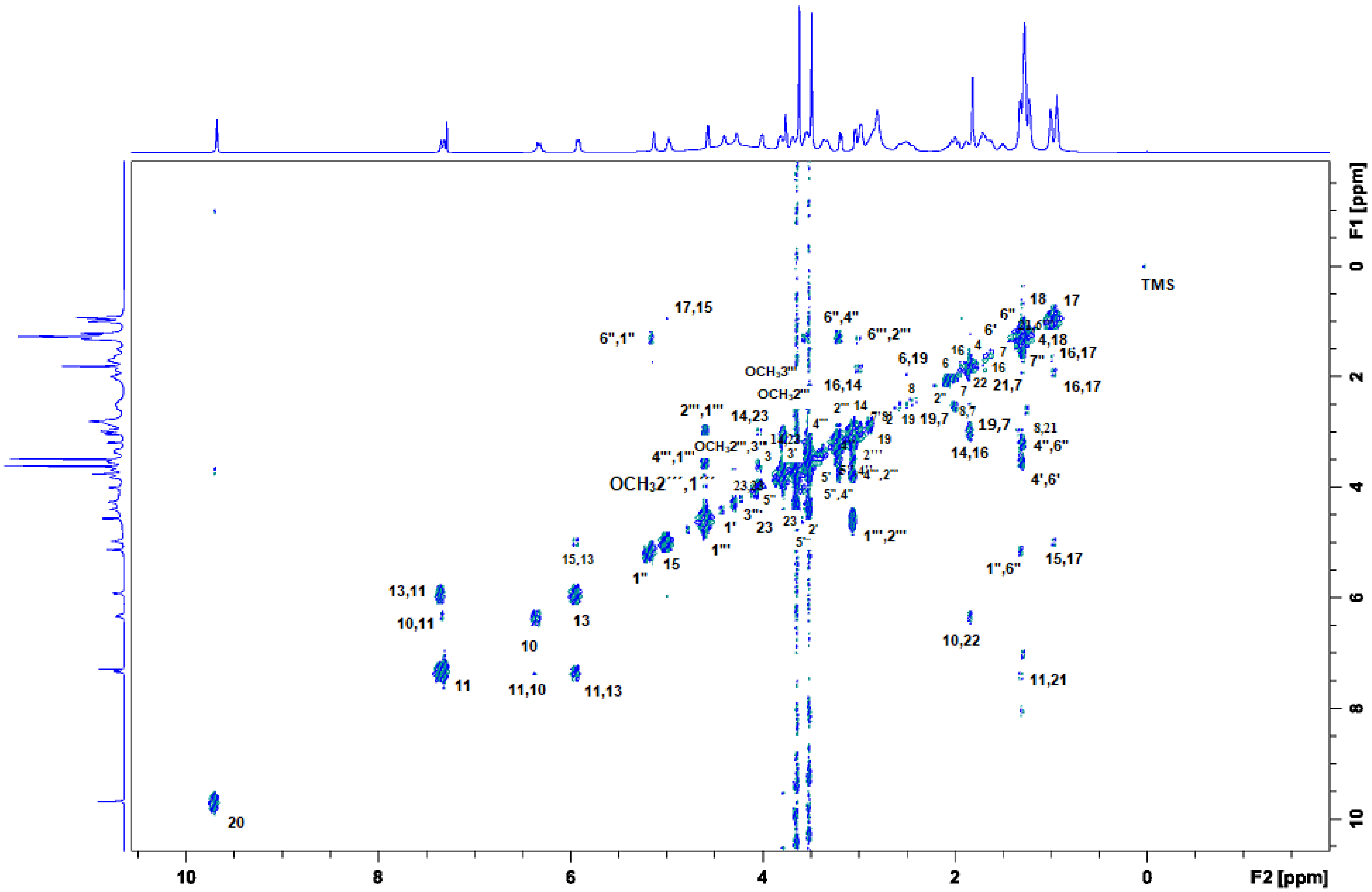

Figure S7: 2D ROESY NMR spectrum of tylosin A in $CDCl_3$.

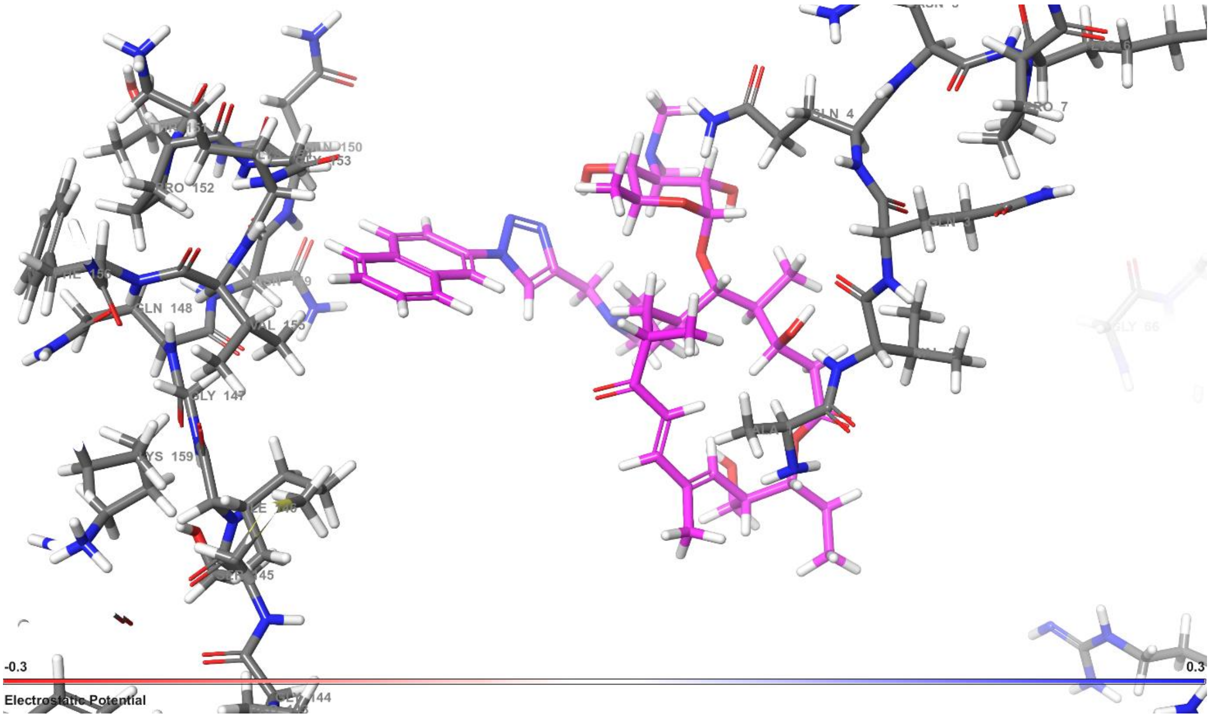

Figure S8: Interactions between (**1**) (pink) and the amino acids from chain B from *E. coli* ribosome.

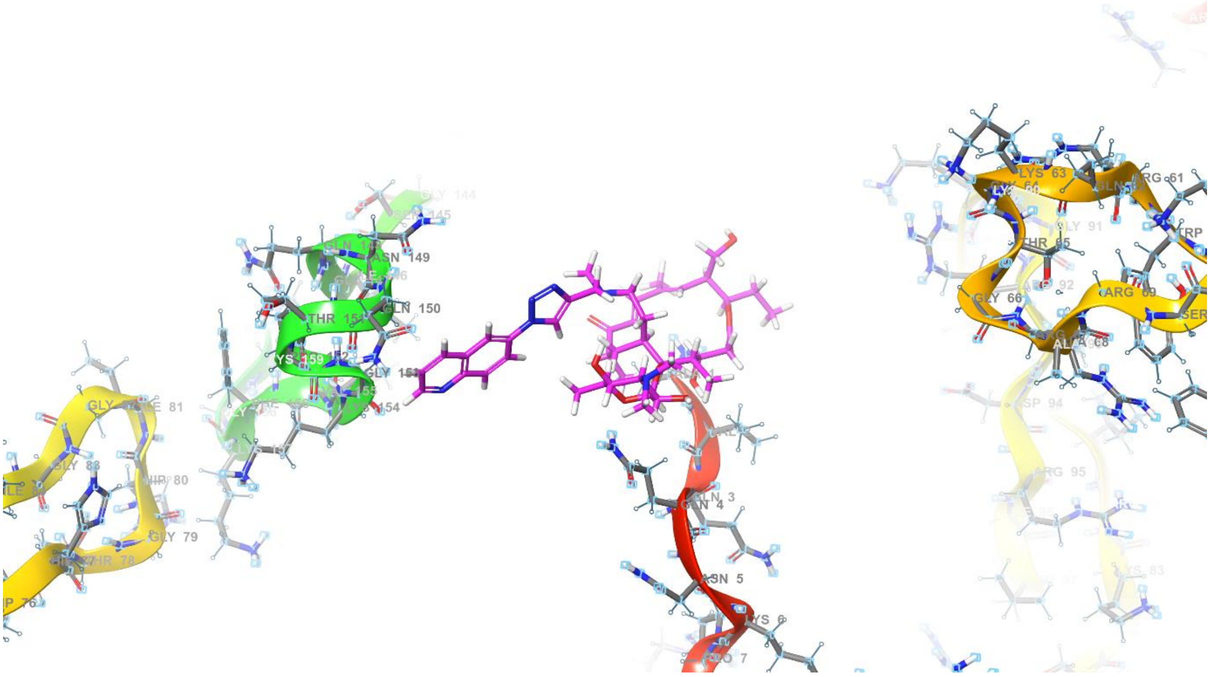

Figure S9: Interactions between (**2**) (pink) and the amino acids from chain B from *E. coli* ribosome.

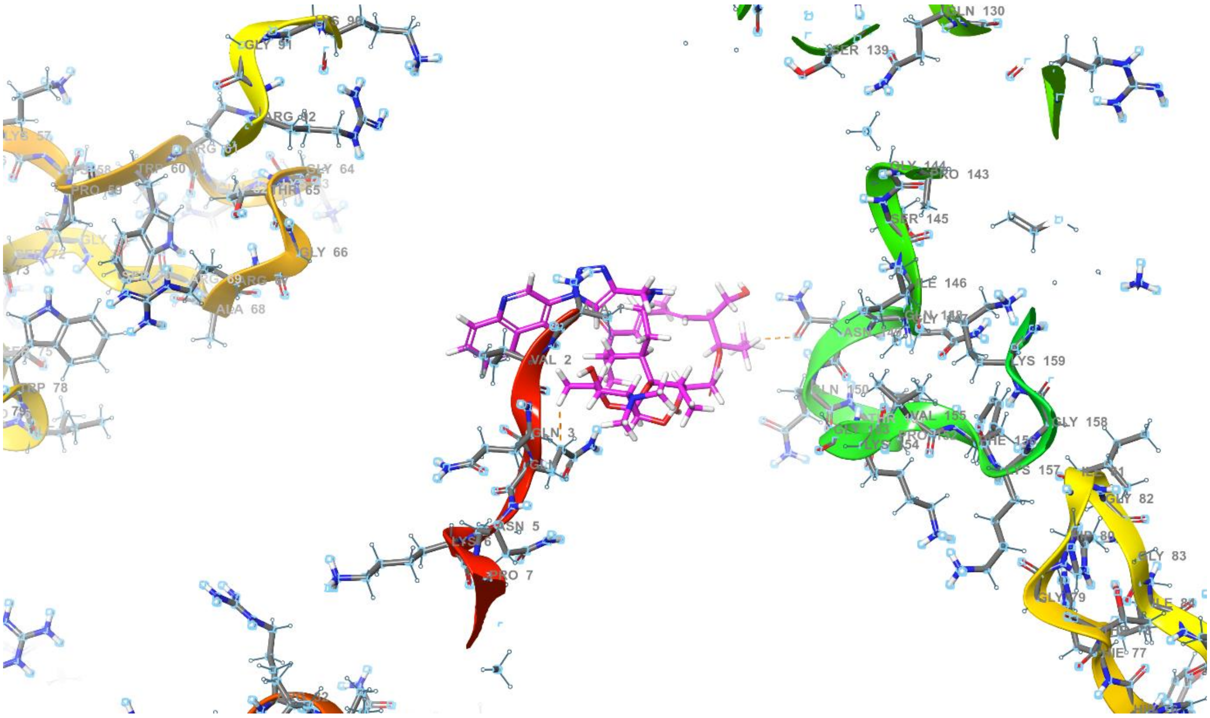

Figure S10: Interactions between (**3**) (pink) and the amino acids from chain B from *E. coli* ribosome.

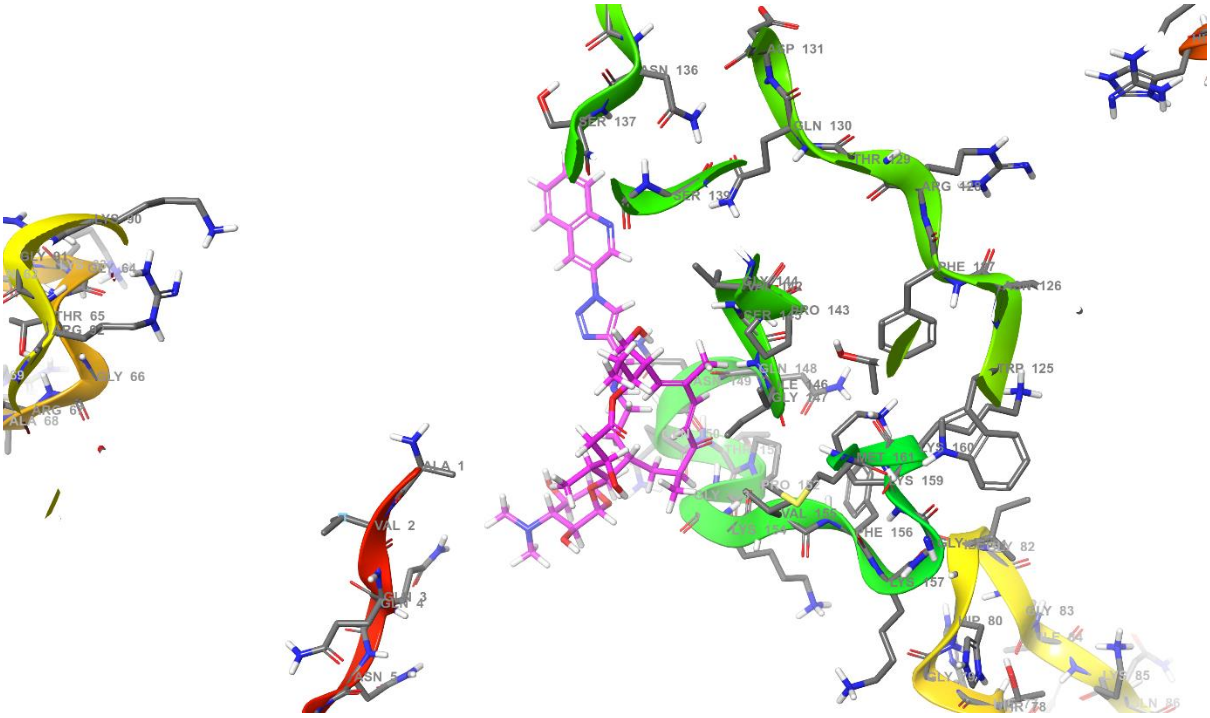

Figure S11: Interactions between (**4**) (pink) and the amino acids from chain B from *E. coli* ribosome

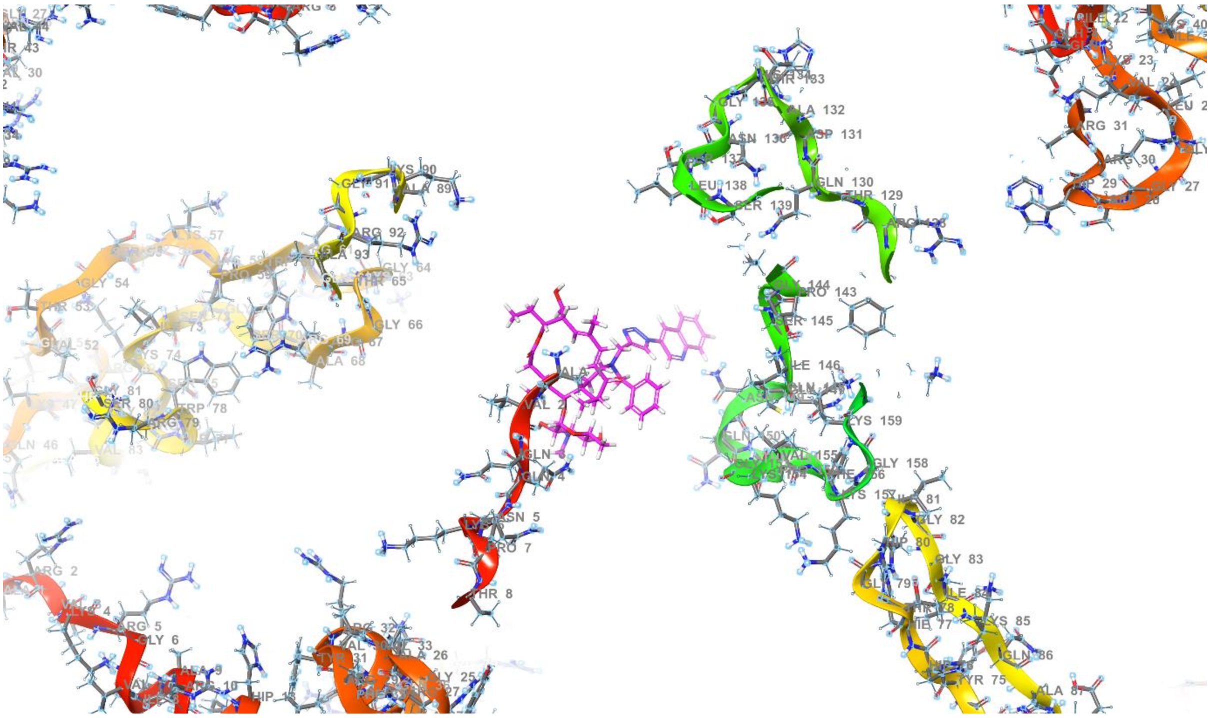

Figure S12: Interactions between (**5**) (pink) and the amino acids from chain B from *E. coli* ribosome

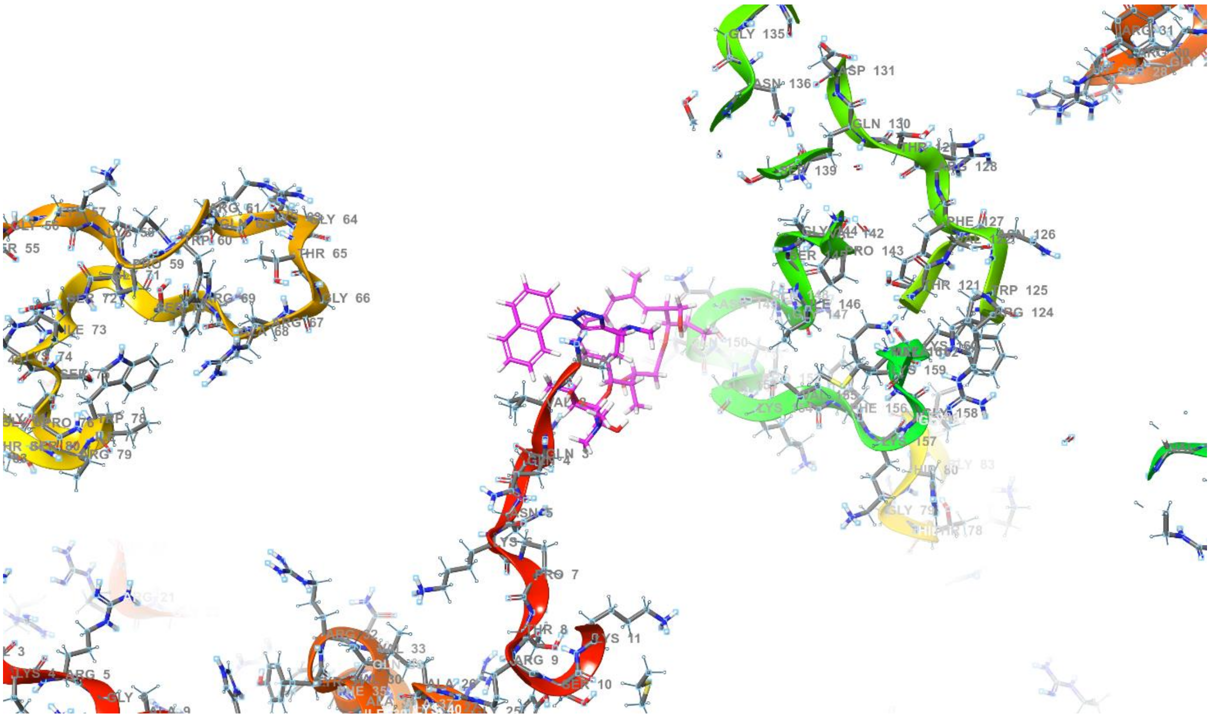

Figure S13: Interactions between (**6**) (pink) and the amino acids from chain B from *E. coli* ribosome.

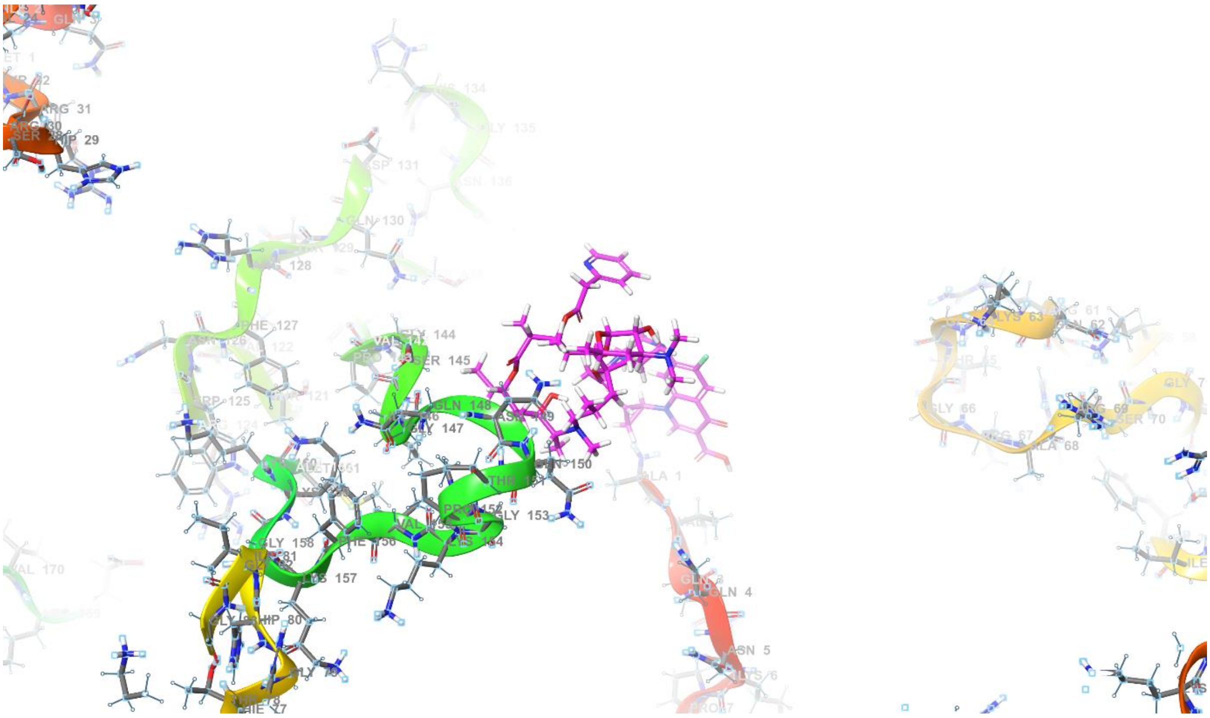

Figure S14: Interactions between (**7**) (pink) and the amino acids from chain B from *E. coli* ribosome.

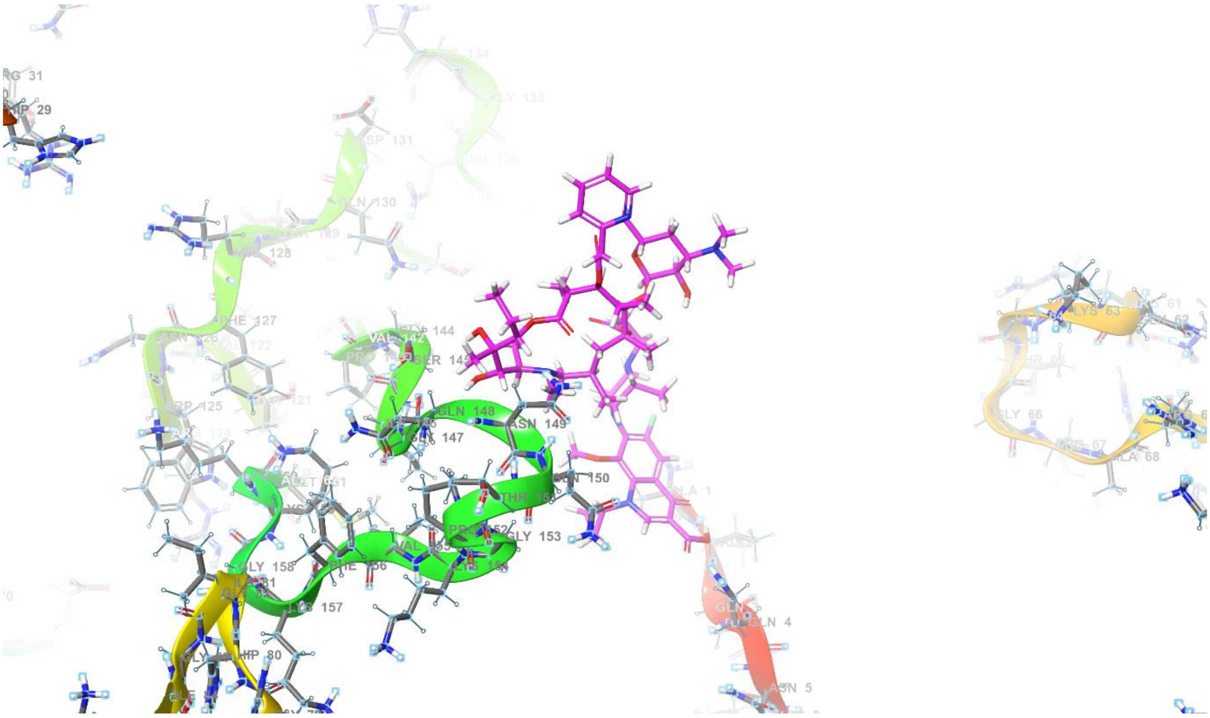

Figure S15: Interactions between (**8**) (pink) and the amino acids from chain B from *E. coli* ribosome.

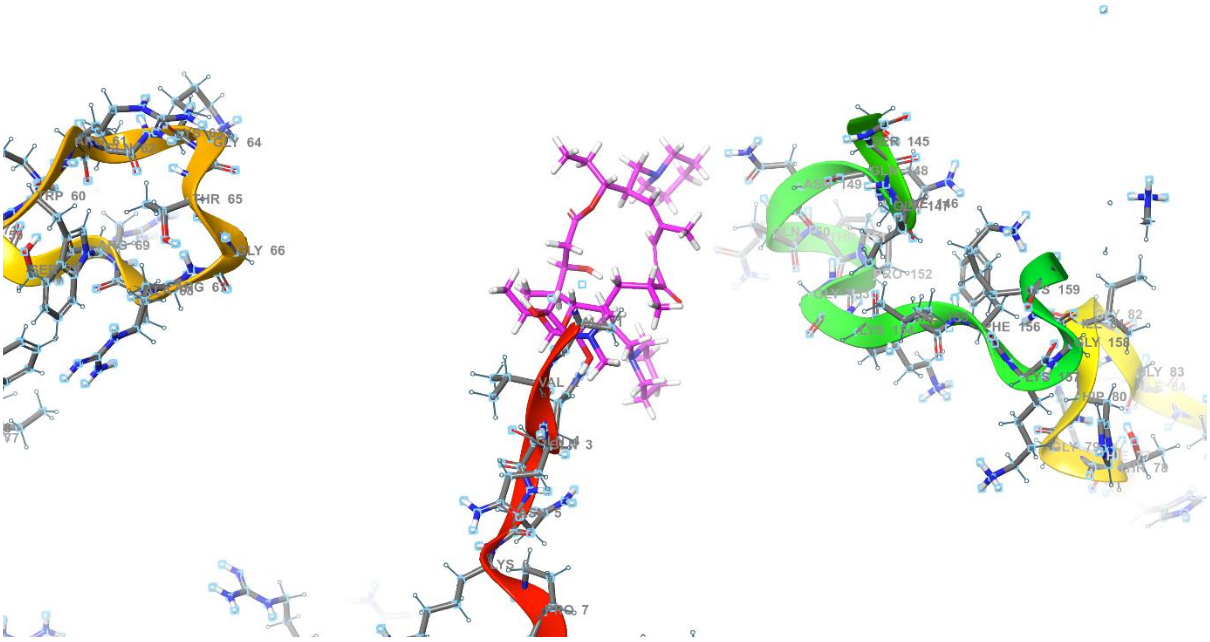

Figure S16: Interactions between tildipirosin (pink) and the amino acids from chain B from *E. coli* ribosome.

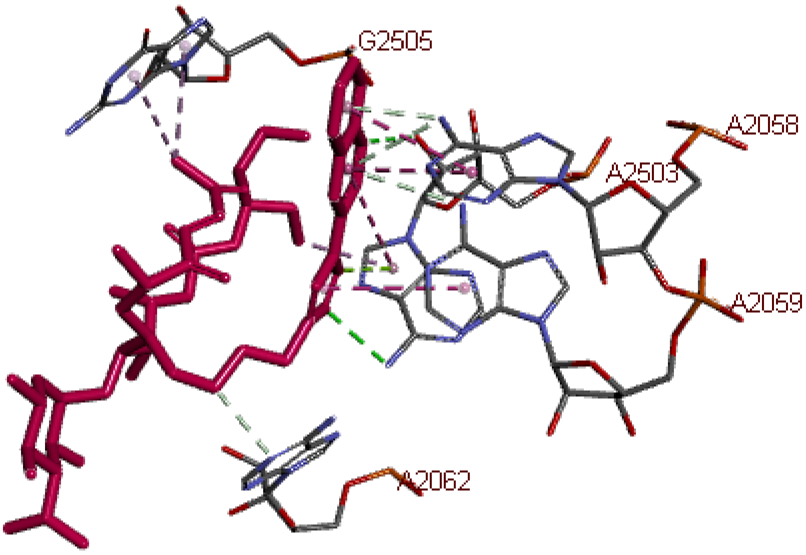

Figure S17: Interactions between (**3**) (red) and the nucleotides from the binding site from *E. coli* ribosome.

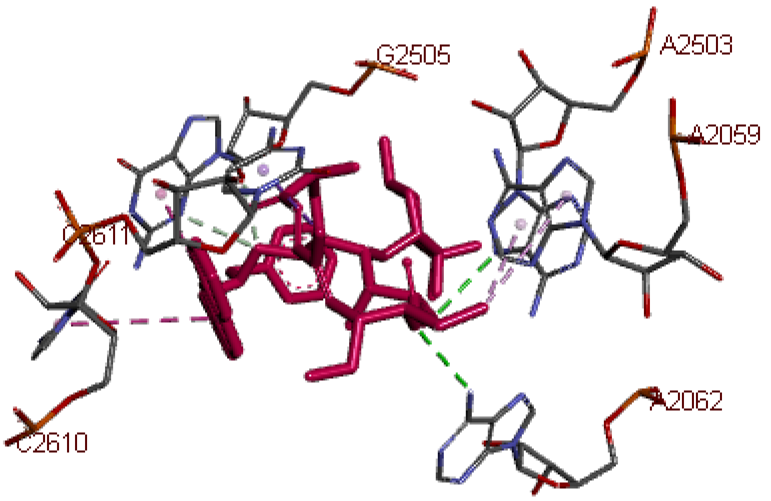

Figure S18: Interactions between (**5**) (red) and the nucleotides from the binding site from *E. coli* ribosome.

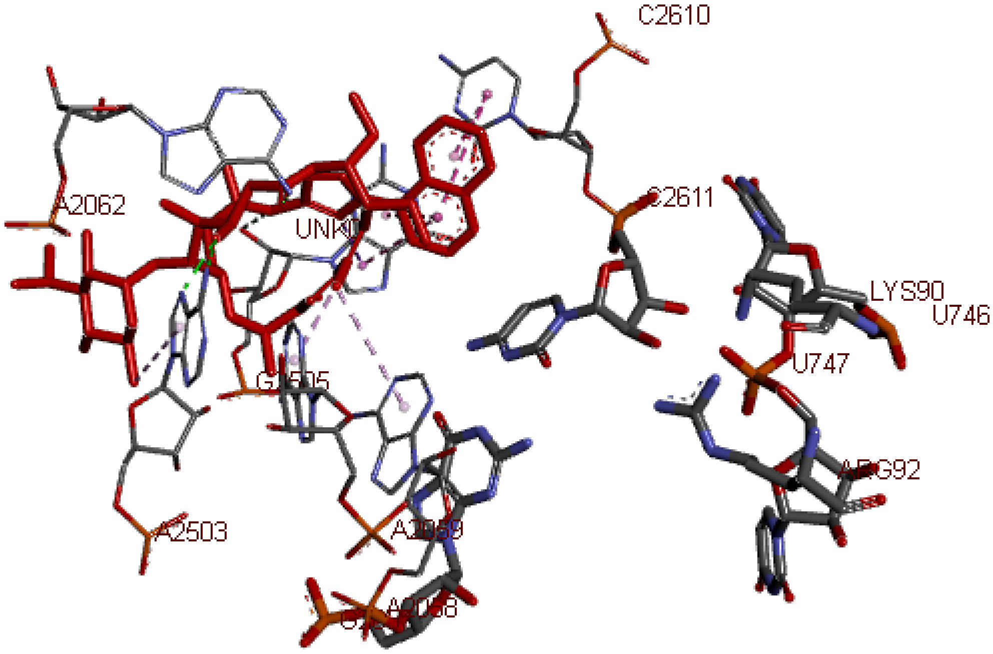

Figure S19: Interactions between (**6**) (red) and the nucleotides from the binding site from *E. coli* ribosome.

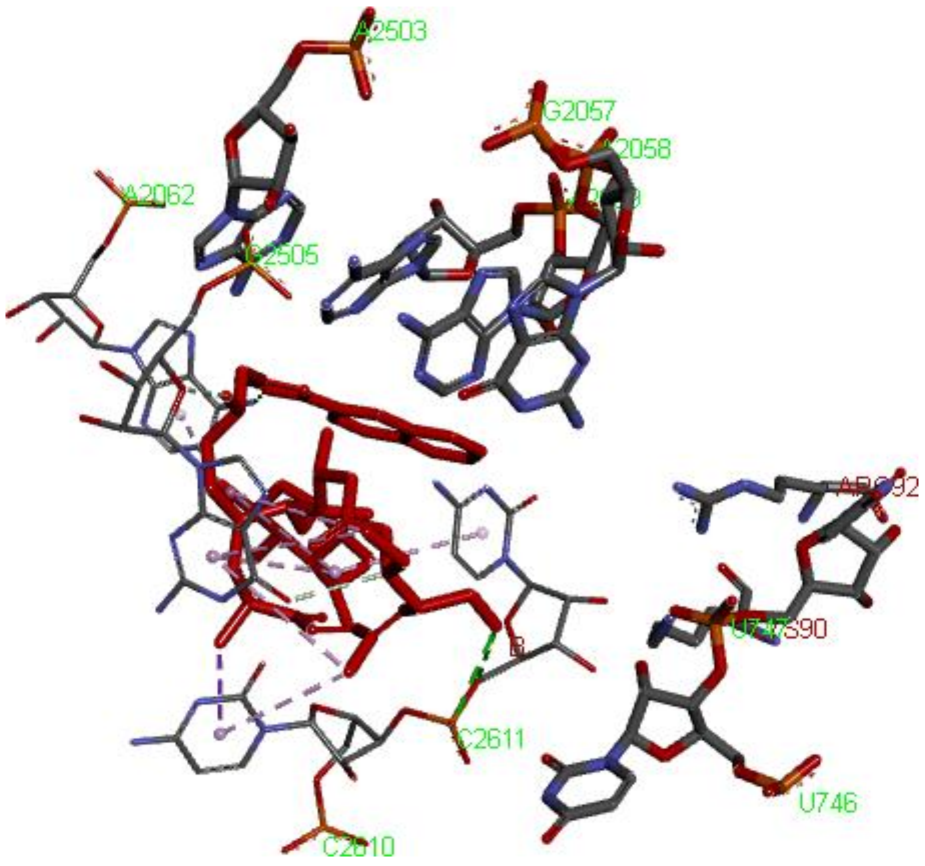

Figure S20: Interactions between (**2**) (red) and the nucleotides from the binding site from *E. coli* ribosome.

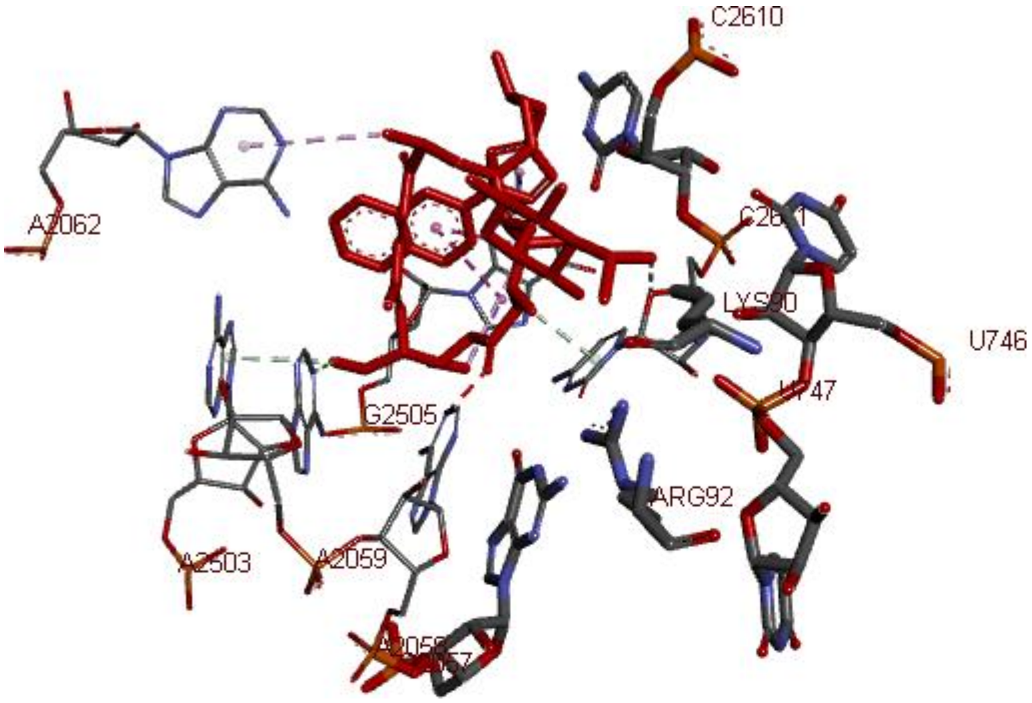

Figure S21: Interactions between (**4**) (red) and the nucleotides from the binding site from *E. coli* ribosome.

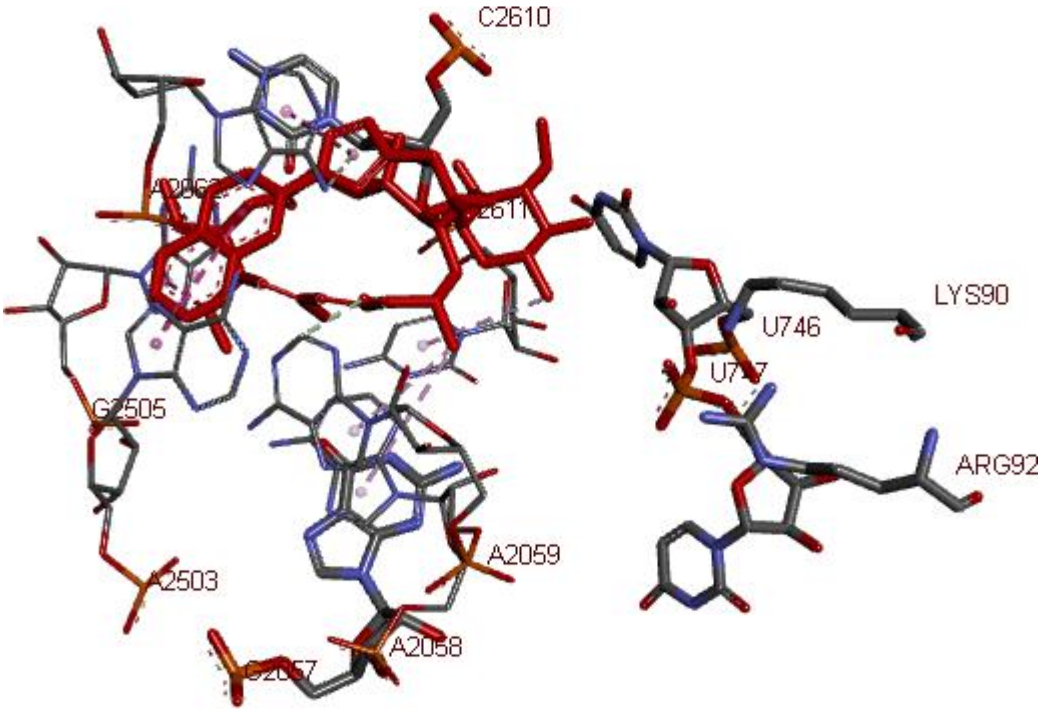

Figure S22: Interactions between (**1**) (red) and the nucleotides from the binding site from *E. coli* ribosome.

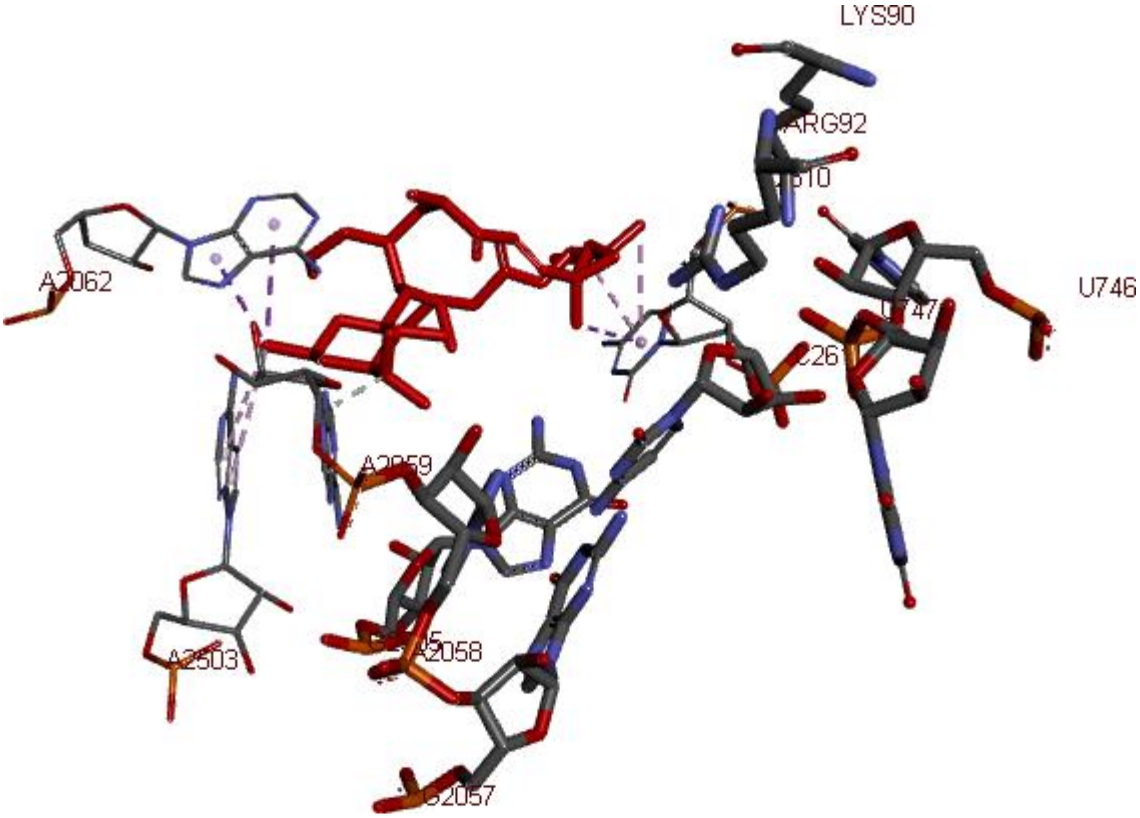

Figure S23: Interactions between rosaramicin (red) and the nucleotides from the binding site from *E. coli* ribosome.

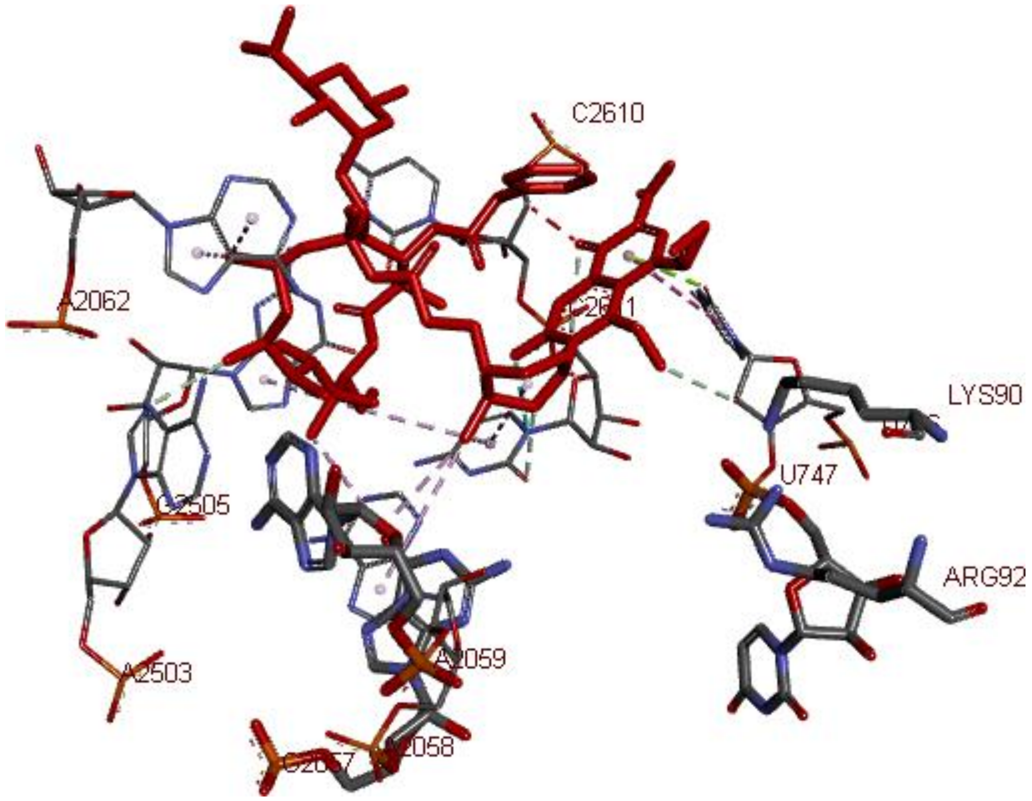

Figure S24: Interactions between (**8**) (red) and the nucleotides from the binding site from *E. coli* ribosome.

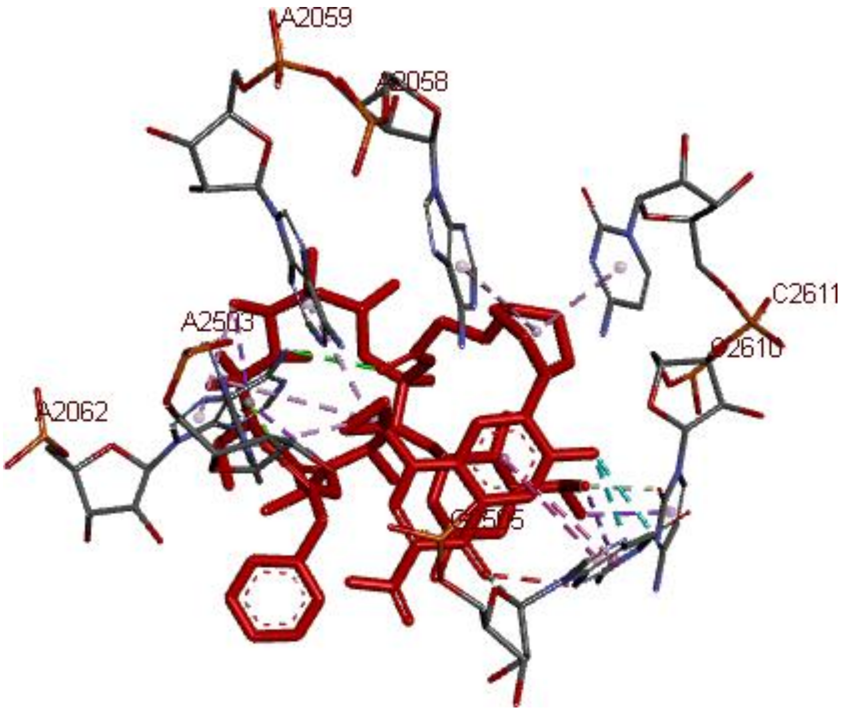

Figure S25: Interactions between (**7**) (red) and the nucleotides from the binding site from *E. coli* ribosome.

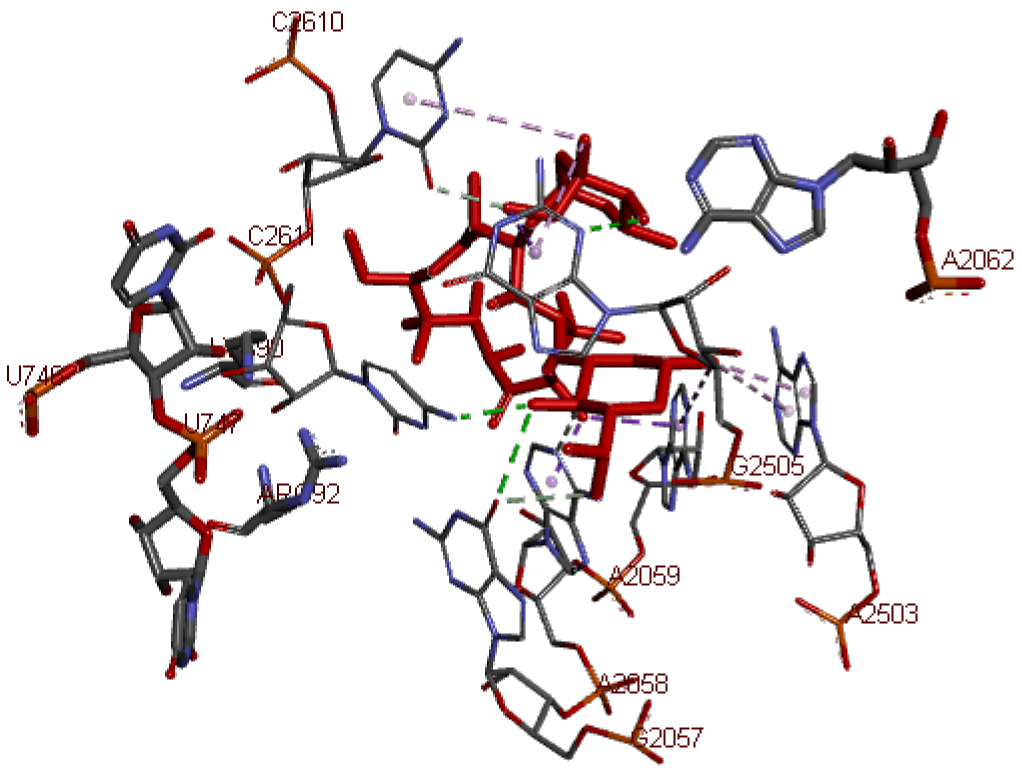

Figure S26: Interactions between erythromycin A (red) and the nucleotides from the binding site from *E. coli* ribosome.

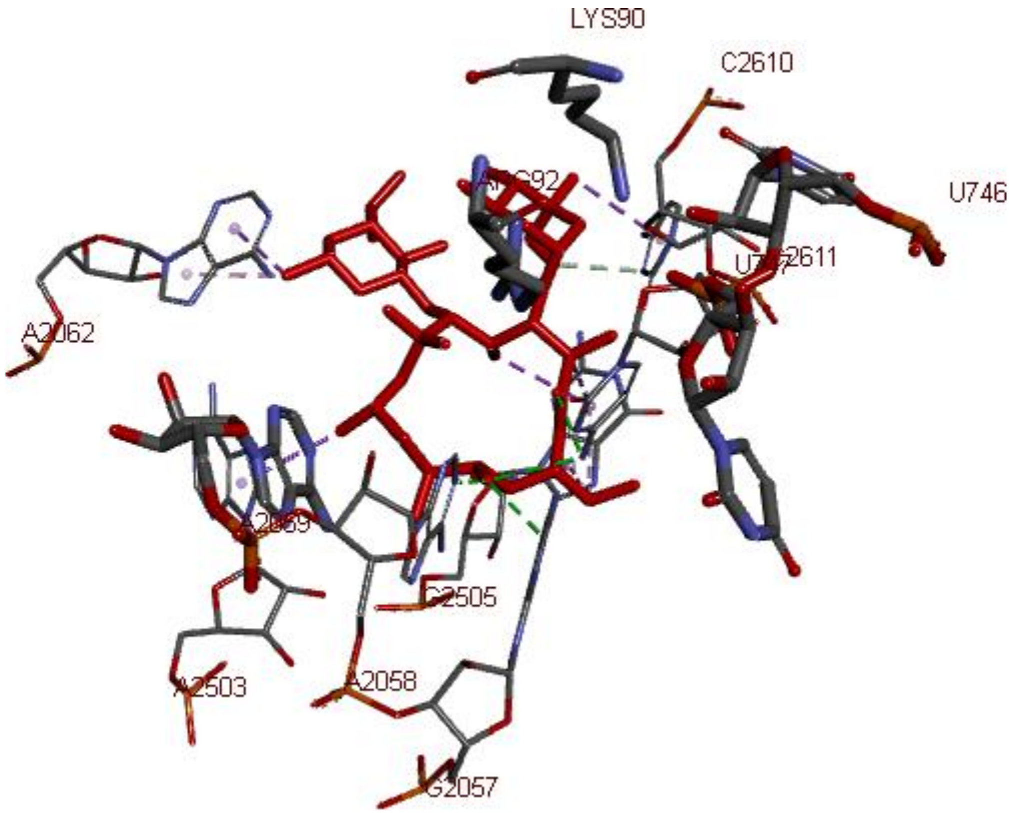

Figure S27: Interactions between erythromycin B (red) and the nucleotides from the binding site from *E. coli* ribosome.

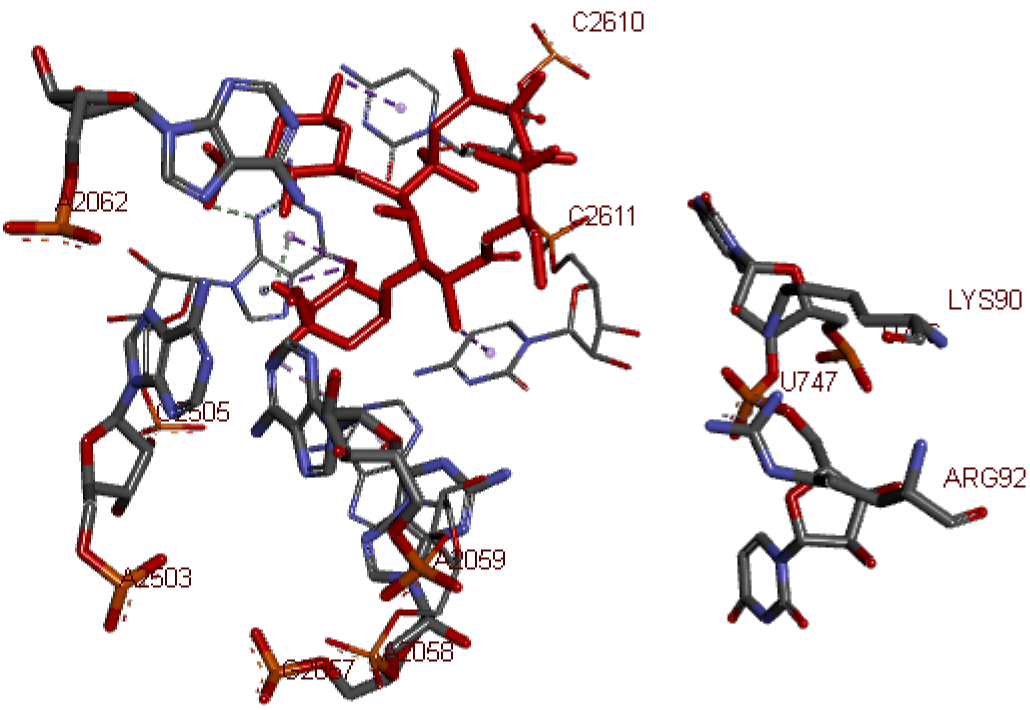

Figure S28: Interactions between erythromycin C (red) and the nucleotides from the binding site from *E. coli* ribosome.

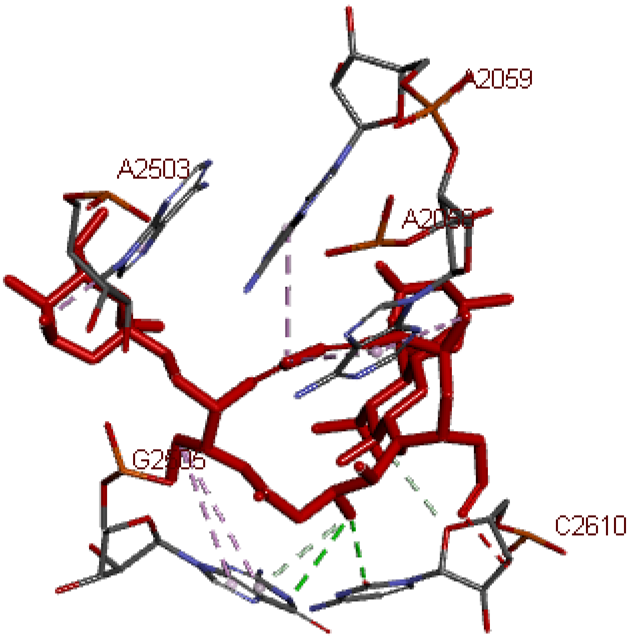

Figure S29: Interactions between tylosin A (red) and the nucleotides from the binding site from *E. coli* ribosome.

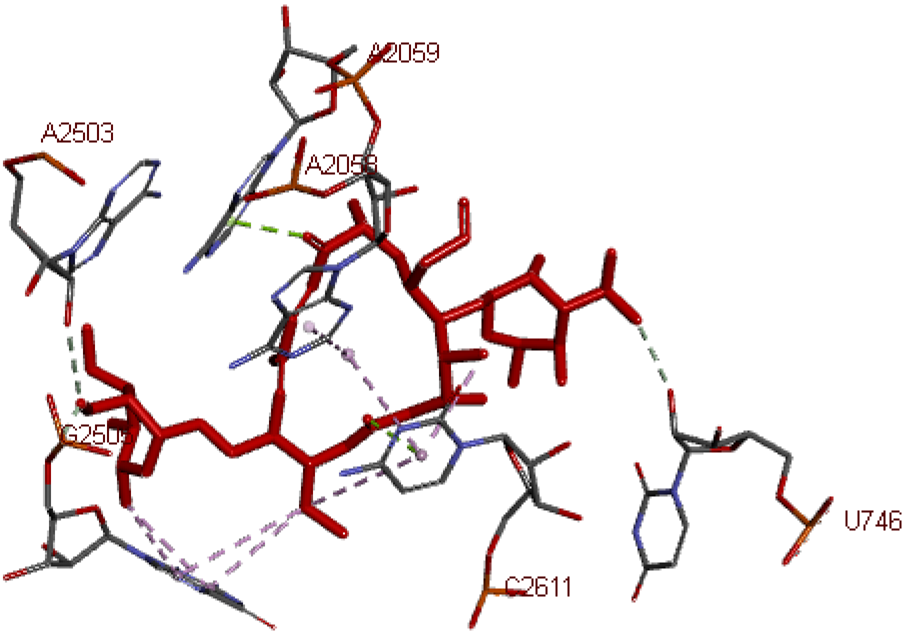

Figure S30: Interactions between tylosin B (red) and the nucleotides from the binding site from *E. coli* ribosome.
